# NGS and phenotypic ontology-based approaches increase the diagnostic yield in syndromic retinal diseases

**DOI:** 10.1007/s00439-021-02343-7

**Published:** 2021-08-26

**Authors:** I. Perea-Romero, F. Blanco-Kelly, I. Sanchez-Navarro, I. Lorda-Sanchez, S. Tahsin-Swafiri, A. Avila-Fernandez, I. Martin-Merida, M. J. Trujillo-Tiebas, R. Lopez-Rodriguez, M. Rodriguez de Alba, I. F. Iancu, R. Romero, M. Quinodoz, H. Hakonarson, Blanca Garcia-Sandova, P. Minguez, M. Corton, C. Rivolta, C. Ayuso

**Affiliations:** 1 Department of Genetics, Health Research Institute-Fundación Jiménez Díaz University Hospital, Universidad Autónoma de Madrid (IIS-FJD, UAM), Madrid, Spain; 2 Center for Biomedical Network Research on Rare Diseases (CIBERER), Instituto de Salud Carlos III, Madrid, Spain; 3 Institute of Molecular and Clinical Ophthalmology Basel (IOB), Basel, Switzerland; 4 Department of Ophthalmology, University of Basel, Basel, Switzerland; 5 Department of Genetics and Genome Biology, University of Leicester, Leicester, UK; 6 Center for Applied Genomics, Children’s Hospital of Philadelphia, Philadelphia, PA USA; 7 Division of Human Genetics, Children’s Hospital of Philadelphia, Philadelphia, PA USA; 8 Department of Pediatrics, Perelman School of Medicine, University of Pennsylvania, Philadelphia, PA USA; 9 Department of Ophthalmology, Health Research Institute-Fundación Jiménez Díaz University Hospital, Universidad Autónoma de Madrid (IIS-FJD, UAM), Madrid, Spain

## Abstract

Syndromic retinal diseases (SRDs) are a group of complex inherited systemic disorders, with challenging molecular underpinnings and clinical management. Our main goal is to improve clinical and molecular SRDs diagnosis, by applying a structured phenotypic ontology and next-generation sequencing (NGS)-based pipelines. A prospective and retrospective cohort study was performed on 100 probands with an a priori diagnosis of non-Usher SRDs, using available clinical data, including Human Phenotype Ontology annotation, and further classification into seven clinical categories (ciliopathies, specific syndromes and five others). Retrospective molecular diagnosis was assessed using different molecular and bioinformatic methods depending on availability. Subsequently, uncharacterized probands were prospectively screened using other NGS approaches to extend the number of analyzed genes. After phenotypic classification, ciliopathies were the most common SRD (35%). A global characterization rate of 52% was obtained, with six cases incompletely characterized for a gene that partially explained the phenotype. An improved characterization rate was achieved addressing prospective cases (83%) and well-recognizable syndrome (62%) subgroups. The 27% of the fully characterized cases were reclassified into a different clinical category after identification of the disease-causing gene. Clinical-exome sequencing is the most appropriate first-tier approach for prospective cases, whereas whole-exome sequencing and bioinformatic reanalysis increases the diagnosis of uncharacterized retrospective cases to 45%, mostly those with unspecific symptoms. Our study describes a comprehensive approach to SRDs in daily clinical practice and the importance of thorough clinical assessment and selection of the most appropriate molecular test to be used to solve these complex cases and elucidate novel associations.

**Supplementary Information:**

The online version contains supplementary material available at 10.1007/s00439-021-02343-7.

## Introduction

Inherited retinal diseases (IRDs) are a clinically and genetically heterogeneous group of ocular rare diseases which, due to the dysfunction of the retina, lead to blindness (Ayuso and Millan 2010; Sundaram et al. 2012). IRDs have an estimated worldwide prevalence of between 1 in 1000 (Hanany et al. 2020) and 1 in 4000 (Ayuso and Millan 2010).

In IRDs, visual impairment can be the sole symptom or can appear together with other features (syndromic forms) such as auditory, nervous, renal, endocrine, musculoskeletal, hepatic, or cardiac anomalies (Wright et al. 2018a). Syndromic retinal diseases (SRDs) are a group of rare and complex inherited systemic diseases representing 20–30% of all IRDs. Some SRDs are well-known and recognizable syndromes, the most common being Usher syndrome (USH, MIM #276901) and other ciliopathies, such as Bardet-Biedl (BBS, MIM #209900), Alström (ALMS, MIM #203800), Joubert (JBTS, MIM #213300), and Senior–Løken (SLSN, MIM #266900) syndromes. However, SRDs also include other rare non-ciliary syndromes related to different cellular components (Werdich et al. 2014) such as Golgi apparatus-related disorders (Cohen syndrome, COH, MIM #216550), endoplasmic reticulum-associated disorders (Wolfram syndrome, WFS, MIM #222300), lysosomal storage disorders (Platt et al. 2018), peroxisome biogenesis disorders (PBD) (Argyriou et al. 2016), and mitochondrial disorders (Kearns–Sayre syndrome, KSS, MIM #530000). In clinical practice, however, the high clinical heterogeneity that patients usually present complicates phenotypic classification into well-defined syndromes, making diagnosis extremely challenging (Chiang and Trzupek 2015). Moreover, inheritance of these disorders can follow any type of Mendelian pattern or be one of a number of very rare non-Mendelian forms such as oligogenic, digenic, or mitochondrial inheritance (Mockel et al. 2011; Werdich et al. 2014; Gazzo et al. 2016). To date, about 94 known genes have been associated with syndromic retinal dystrophies (The Retinal Information Network, RetNet: https://sph.uth.edu/retnet/; last accessed January 2021).

Next-generation sequencing (NGS) technologies are currently the gold-standard approach for cost-effective genetic analysis in extremely complex syndromic forms (Wright et al. 2015). Their previous application into SRD cases has been proven to improve the diagnostic yield when compared to other traditional, time-consuming genotyping methods (Sanchez-Navarro et al. 2018; Abu Diab et al. 2019). Moreover, whole-exome sequencing (WES) and whole-genome sequencing (WGS) allow further reanalysis and revisions of previously-investigated cases using updated virtual panels, reanalysis with new bioinformatic tools (Wright et al. 2018b) and strategies using ontologies, which are useful for conducting targeted studies (Köhler et al. 2009).

The development of a clinical and genetic pipeline for the precision study of SRDs could help to anticipate additional underlying systemic complications that require periodic surveillance for early detection, management, and treatment. Such a pipeline would have repercussions for reproductive risk assessment, genetic counseling and patient selection for clinical trials of gene-based therapies (Ayuso and Millan 2010; Lee and Garg 2015; Sadagopan 2017).

The main aim of this study is to improve clinical and molecular SRD diagnosis, applying new structured Human Phenotype Ontology (HPO)-based phenotypic and NGS-based pipelines. This research has resulted in the creation of a working protocol for the precision study of SRDs in our institution.

## Materials and methods

### Subjects

Study subjects were recruited through a search of the database of the Genetics Department at the Fundación Jiménez Díaz Hospital (FJD) (Madrid, Spain), which includes a cohort of 4403 families with diverse IRDs referred for genetic testing since 1991 (Perea-Romero et al. 2021). Probands selected for study were required to meet the following inclusion criteria: (1) presumed diagnosis of SRD; (2) retrospective cases with a negative or non-informative genetic testing until December 2017 (*n* = 82), or new prospective cases between January 2018 and October 2020 (*n* = 18). Patients with typical Usher syndromes were excluded from this study.

This research has been approved by the FJD Research Ethics Committee and complies with all the principles of the Declaration of Helsinki and further revisions. All patients, or their legal guardians when necessary, signed a written informed consent form before entering the study.

### Phenotypic data and classification

Clinical examination was made according to previously established criteria and included ophthalmic, physical, and additional examinations as previously described in Piñeiro-Gallego et al. (2012), Sanchez-Navarro et al. (2018) and Galbis-Martínez et al. (2021), as well as self-reported health data. Clinical and family history were reviewed through clinical reports, specific questionnaires, and/or electronic health records for each participant. Presumed diagnosis at the time of referral for genetic testing was also considered.

Probands were classified into seven different major a priori phenotypic groups using a modified categorization from Sanchez-Navarro et al. (2018). The diagnostic criteria used during phenotypic classification are described in Supplementary Table S1.

Probands with a well-recognizable syndrome were organized under two disease groups: (1) ciliopathies (BBS, ALMS, JBTS, or SLSN) or suspicion of ciliopathy (ciliopathies-like), due to the presence of most ciliopathy-associated signs such as postaxial polydactyly, molar tooth sign on magnetic resonance imaging, or polycystic kidney disease, among others; and (2) other specific SRDs, including known or presumed clinical entities (Supplementary Table S1).

The remaining unclassified cases were grouped according to the major extra-ocular symptoms using ontology terms derived from HPO annotation (Köhler et al. 2009) and classified as (3) hearing loss (HL) and/or neurodevelopmental disorders, (4) neuropathy/myopathy and/or suspicion of mitochondrial DNA (mtDNA) disorder, (5) skeletal disorders, (6) other unspecific symptoms, and (7) unclassified due to the lack of additional clinical information. These five categories are summarized in the Supplementary Table S1.

### Molecular screening and bioinformatic analysis

Over the study period, different molecular strategies were used in genetic testing and, in some cases, the same proband had been studied by several methods. A summary of the molecular analysis performed in our cohort is described in Supplementary Fig. S1.

The 18 prospective probands were mostly analyzed as a first-tier analysis using commercial clinical exome sequencing (CES) approaches: TruSight One Sequencing Panel kit (Illumina, San Diego, CA, USA) or Clinical Exome Solution (Sophia Genetics, Boston, MA, USA). Libraries were prepared following the instructions of each manufacturer and sequenced on a NextSeq500 platform (Illumina) (Martin-Merida et al. 2019). Bioinformatic analysis for single nucleotide variants (SNVs) was performed using the Illumina software from BaseSpace coupled with the VariantStudio v.3.0.12 software, or SOPHiA DDM platform (Sophia Genetics), respectively. Variant filtering and prioritization were based on read depth ≥ 20, frequency of the alternative allele > 20%, and minor allelic frequency (MAF) in Genome Aggregation Database (gnomAD < 0.02). Potentially pathogenic variants were prioritized for a 136-gene subpanel of non-syndromic and syndromic IRD genes; if a negative result was obtained, CES data were later prioritized with an expanded virtual panel of up to 377 genes (Supplementary Table S2). Copy number variant (CNV) detection was carried out using the CoNVading software (Johansson et al. 2016) or SOPHiA DDM platform, respectively.

The 82 probands recruited before 2018 had been unsuccessfully screened using different approaches over time including classical molecular genetics, aCGH, and/or a customized 121-gene targeted NGS approach (Castro-Sánchez et al. 2015; Sanchez-Navarro et al. 2018).

After these preliminary analyses, uncharacterized probands (*n* = 79) were additionally screened using exome sequencing to extend the number of analyzed genes, including the above described CES approach.

Afterwards, 34 probands were analyzed by means of WES, as well as three more cases directly sequenced by this NGS technique. WES was carried out mostly using the Agilent SureSelect Human All Exon V5 kit and sequenced on an Illumina HiSeq2500 (Tatour et al. 2017). First, potentially pathogenic variants were prioritized in the extended 447-gene subpanel of non-syndromic and syndromic IRD, optic atrophy, and associated genes (Supplementary Table S2) using an in-house pipeline described in Supplementary Table S3. For gene discovery purposes, the WES data of 26 probands were analyzed using a hypothesis-free approach, including homozygosity mapping in probands with family history of consanguinity using the AutoMap tool (Quinodoz et al. 2021). Variant prioritization was performed as described in Supplementary Table S3. Re-analysis of NGS data from uncharacterized probands was performed every 2 years using updated in-house pipelines (Supplementary Table S3).

Finally, in five retrospective cases with suspected mitochondrial disorder, mtDNA sequencing was carried out using the QIAseq targeted Human mitochondrial DNA kit (QIAGEN GMBH) on an Illumina MiniSeq. Bioinformatic analysis, sequence alignment, and variant annotation were performed using the GeneGlobe Data Analysis Center (QIAgen). Variant prioritization was performed by integrating data from the MITOMAP database (https://www.mitomap.org/) using a custom script. The mtDNA haplogroup was established using HaploGrep (https://haplogrep.uibk.ac.at/).

### Variant classification and validation studies

Candidate variants obtained were analyzed in detail and filtered according to disease databases such as OMIM (https://www.omim.org/), ClinVar (https://www.ncbi.nlm.nih.gov/clinvar/), HGMD Professional 2020.4 and/or Leiden Open Variation Databases (LOVD, https://www.lovd.nl/). Variant classification was carried out using a 5-class system (class 1 for benign to class 5 for pathogenic variants) following the recommendations of the American College of Medical Genetics and Genomics (ACMG) (Richards et al. 2015) and the European Society of Human Genetics (ESHG) criteria (Matthijs et al. 2016).

Sanger sequencing was performed to validate all predicted variants classified as class 4 and 5 variants and the variants of uncertain significance (VUS), or class 3, in a gene relevant to the clinical phenotype. CNVs were validated by means of a custom aCGH modified from the arrEYE platform described in Van Cauwenbergh et al. (2017), allowing for a high-resolution study of CNVs in 125 IRD-related genes. Homozygosity was confirmed in all homozygous variants using CNV detection tools in the CES or WES data. Segregation analysis for SNVs and CNVs was also performed when DNA samples were available for affected and unaffected family members.

A proband was considered to have been characterized when any of these situations occurred: (1) identification of potentially biallelic variants classified as class 3, 4, or 5 in a recessive gene in homozygosis or a class 3 accompanying a class 4 or 5 variant; (2) identification of a heterozygous allele for a class 4 or 5 variant in a dominant gene; or (3) identification of a hemizygous class 4 or 5 variant in an X-linked gene. Furthermore, variants were required to explain the phenotype totally or partially and segregate in the pedigree (when this analysis was possible).

### Statistical analysis

To test the improvement of the proposed approaches in the rate of characterization achieved, a Chi-squared test was performed and *p*-values lower than 0.05 were considered significant.

## Results

### Cohort description and phenotypic a priori classification

Here, we analyzed a cohort of 100 uncharacterized probands (54 females and 46 males) presenting an SRD.

A total of 82 uncharacterized cases were retrospectively identified from our electronic database from over 26 years (from 1991 to 2017) of studies. These cases had been unsuccessfully screened using different approaches over time, including classical genetic tests, aCGH, and/or a customized 121-gene targeted NGS approach, and finally new technologies including CES and/or WES. In the 26 uncharacterized cases after WES analysis, reanalysis was performed by means of new bioinformatic approaches, including reannotation of old data (Supplementary Table S3).

Eighteen probands were prospectively recruited at the FJD over the last 3-year period (from 2018 to 2020) after being referred for genetic testing and were further screened by CES approaches as first-tier analysis (Supplementary Fig. S1).

**Fig. 1:**
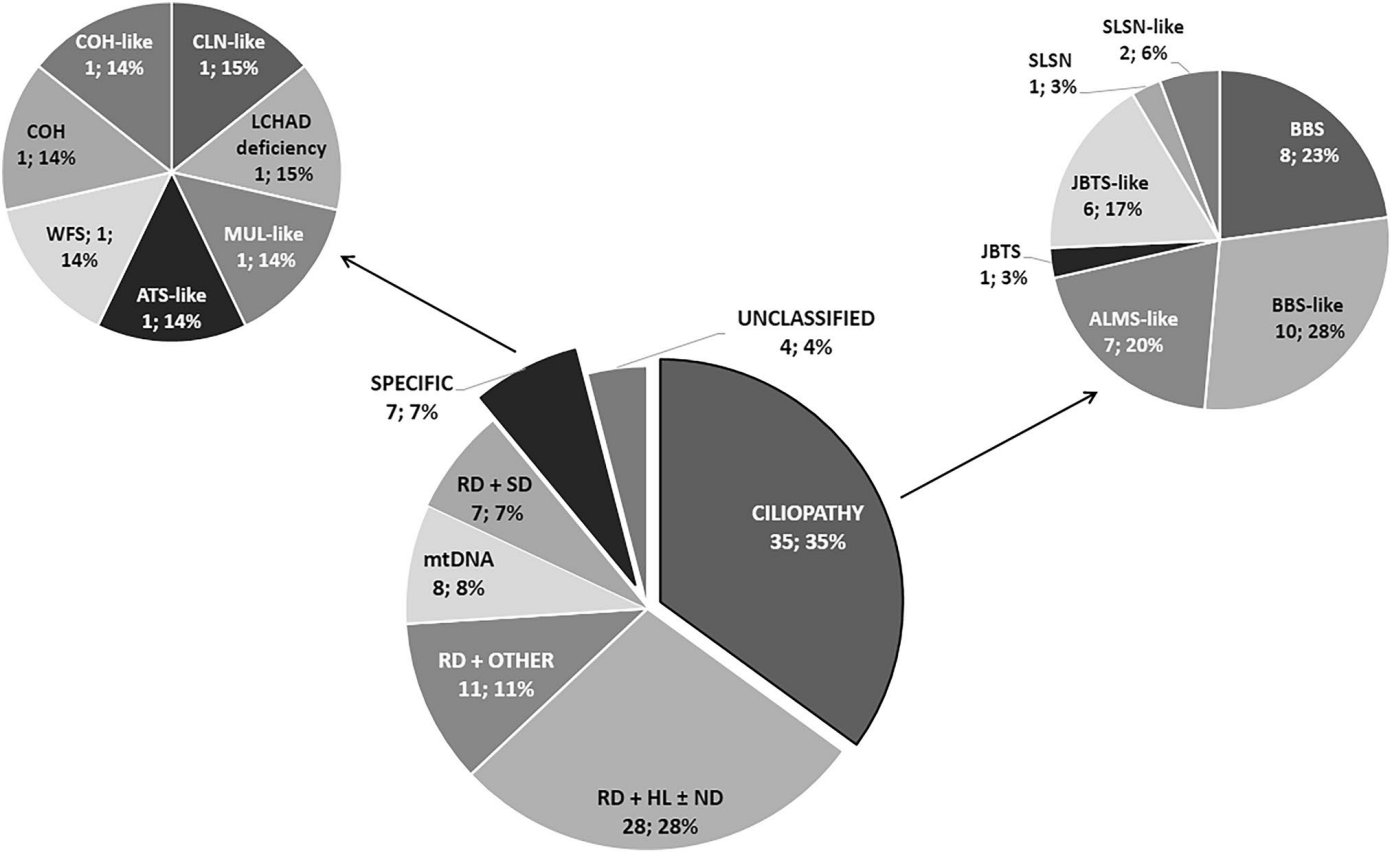
Presumed a priori diagnosis and phenotypic classification. All the cases were classified according to their phenotype into seven different categories: (i) suspicion of ciliopathy or ciliopathy-like (CILIOPATHY); (ii) suspicion of specific rare disease (SPECIFIC); (iii) RD + hearing loss and/or neurodevelopmental disorder (RD + HL ± ND); (iv) RD + neuropathy or myopathy or a suspicion of mitochondrial DNA disorder (mtDNA); (v) RD + skeletal disorder (RD + SD); (vi) RD + other (RD + OTHER); and (vii) SRDs unclassified without clinical information (UNCLASSIFIED). Subsequently, the patients with suspicion of ciliopathy or ciliopathy-like and specific rare disease were divided into different clinical entities using the pipeline provided in the Supplementary Table S1. *ALMS* Alström syndrome, *ATS* Alport syndrome, *BBS* Bardet-Biedl syndrome, *CLN* ceroid lipofuscinosis, neuronal, *COH* Cohen syndrome, *JBTS* Joubert syndrome, *LCHAD deficiency* long-chain 3-hydroxiacyl-CoA dehydrogenase deficiency, *MUL* Mulibrey nanism, *RCD* rod-cone dystrophy, *RD* retinal dystrophy, *SLSN* Senior–Løken syndrome, *SRD* syndromic retinal diseases, *WFS* Wolfram syndrome

After thorough revision of the clinical history, patients were classified following a priori diagnosis of SRD considering the main symptoms and HPO criteria defined in Supplementary Table S1. Probands were split into seven clinical groups (Fig. 1). The most common SRD in our cohort was ciliopathies or ciliopathy-like disorders with 35 probands. Specifically, BBS/BBS-like was the most widely represented phenotype, as half (18/35) of the probands with suspected ciliopathies, followed by ALMS and JBTS with seven probands in each of both well-recognized phenotypes. Furthermore, some specific non-ciliary conditions were also suspected in our cohort, such as COH, WFS, Alport syndrome (ATS, MIM #301050), ceroid lipofuscinosis (CLN, MIM #256730), long-chain 3-hydroxyacyl-CoA dehydrogenase deficiency (LCHAD deficiency, MIM #609016), and Mulibrey nanism (MUL, MIM #253250).

Among the group of 54 probands without a well-known syndrome, HL and/or neurodevelopmental disorders (ND) were the extra-ocular symptoms most frequently associated with retinopathy in half (28/54) of these probands. In 23 out of 28 probands, HL and neurodevelopmental disorders usually presented as isolated systemic findings, while in the remaining five, both appeared together. Eleven probands presented miscellaneous isolated unspecific systemic symptoms (RD + OTHER), such as type I diabetes mellitus, obesity, dysmorphias, diverse congenital malformations, pancytopenia, or neuroendocrine alterations, which impaired classification as a more distinctive retinal syndrome.

**Fig. 2:**
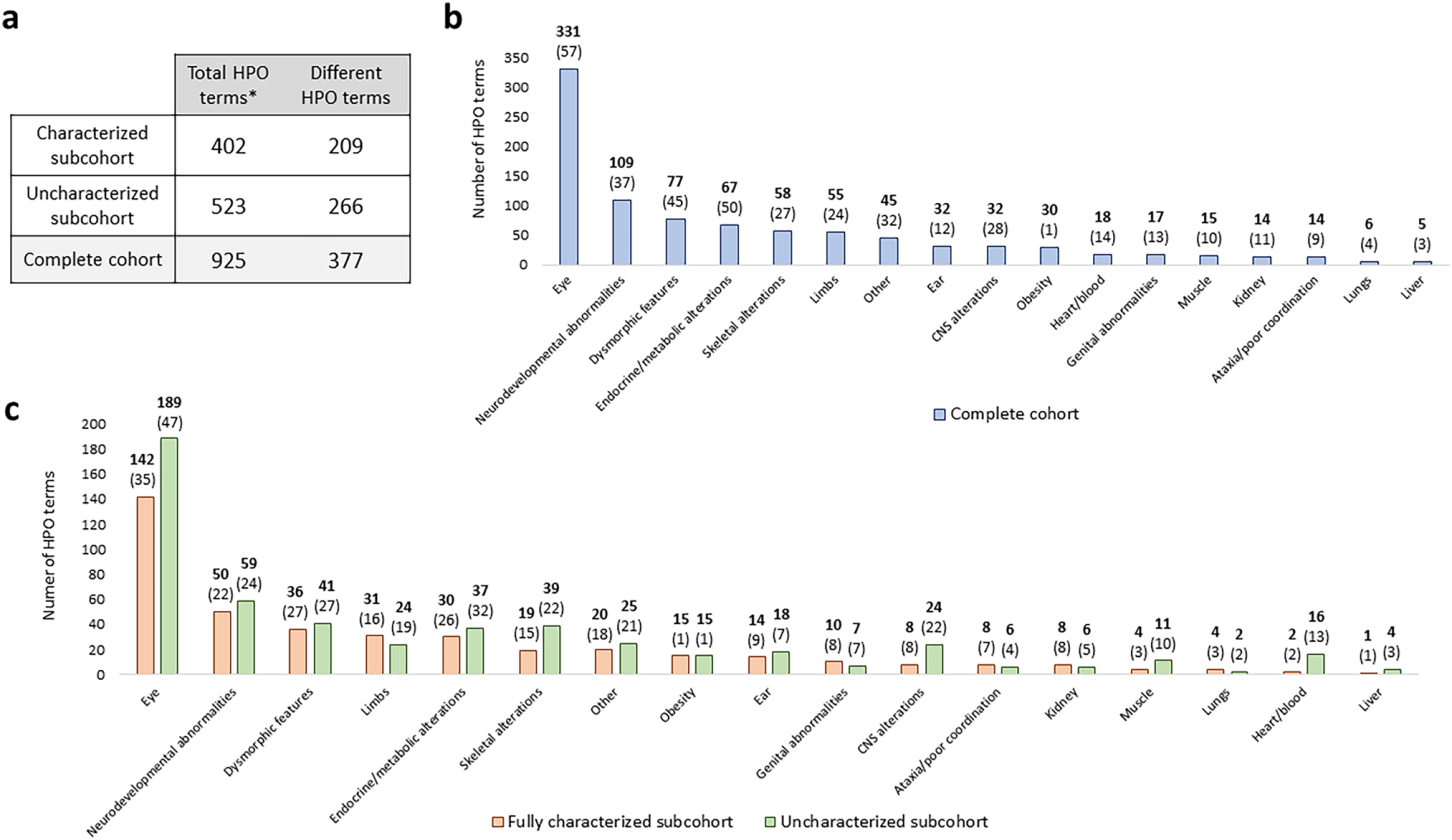
Summary of the HPO terms grouped by system. **a** Total identified and different HPO terms in the complete cohort and the fully characterized and uncharacterized (partially characterized, monoallelic, and negative cases) subcohorts. *The phenotypic terms without HPO annotation (*n* = 19) have been excluded. **b** All the terms identified in the complete cohort were classified. Numbers in bold represent the total HPO terms identified in each category, while the number of different HPO terms appears in brackets. **c** All the terms identified in the fully characterized and the uncharacterized subcohort were classified

A total of 925 HPO terms were annotated in the cohort from 377 different terms. The most common non-ocular ontology system group was the one comprising neurodevelopmental abnormalities-related terms (Supplementary Table S4 and Fig. 2a). There were no significant differences in the distribution of HPO terms between the fully characterized and uncharacterized cases, although there was slight enrichment of terms related to alterations in the central nervous (CNS), musculoskeletal, and cardiovascular systems in the subgroup of uncharacterized patients (Fig. 2b).

### Outcomes and diagnostic yield of genetic testing

**Fig. 3:**
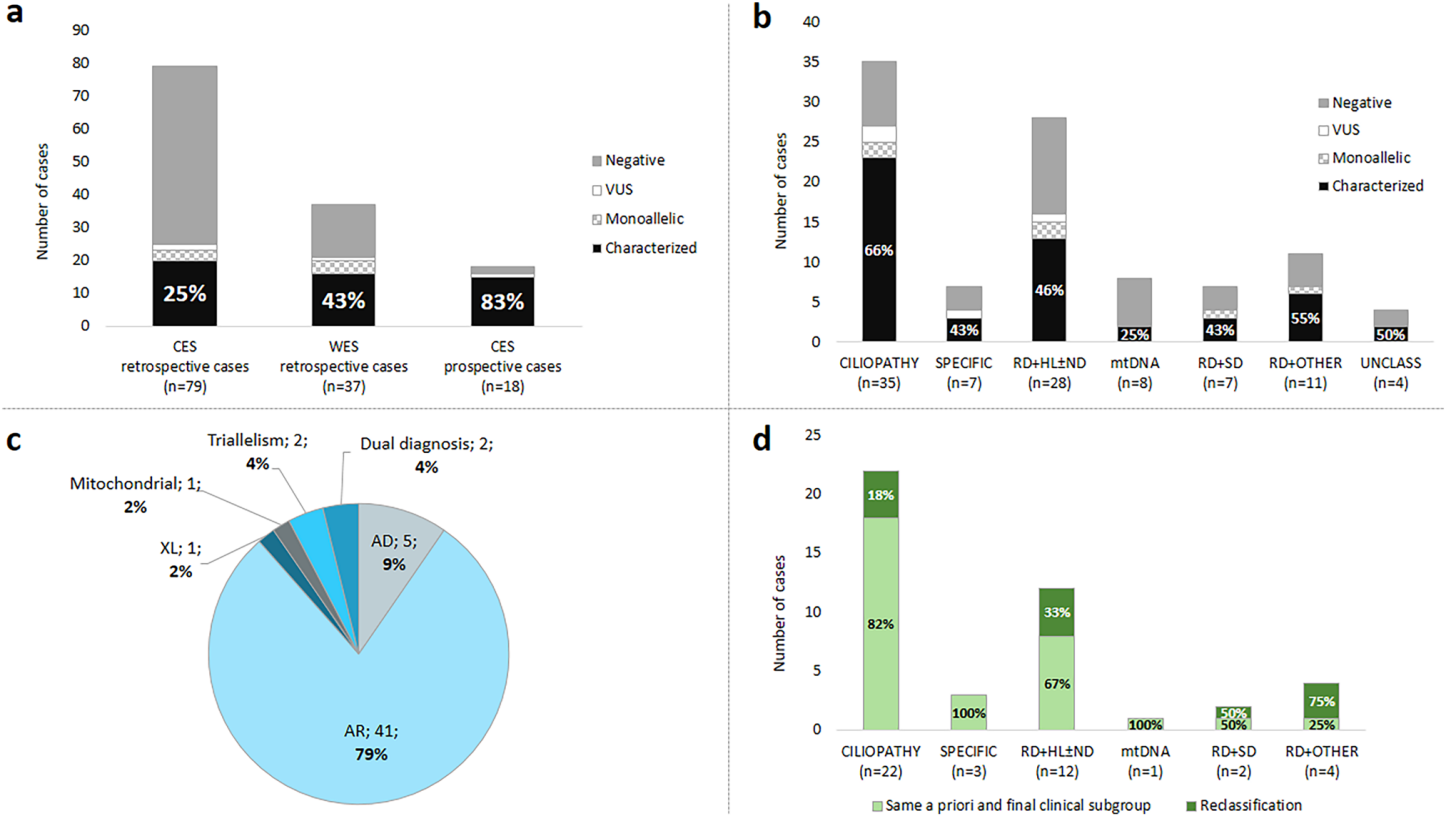
Molecular results. The genetic results were divided considering the a priori clinical groups: (i) suspicion of ciliopathy or ciliopathy-like (CILIOPATHY); (ii) suspicion of specific rare disease (SPECIFIC); (iii) RD + hearing loss and/or neurodevelopmental disorder (RD + HL ± ND); (iv) RD + neuropathy or myopathy or a suspicion of mitochondrial DNA disorder (mtDNA); (v) RD + skeletal disorder (RD + SD); (vi) RD + other (RD + OTHER); and vii) SRDs unclassified without clinical information (UNCLASS). **a** Diagnostic yields of molecular testing depending on the NGS technology carried out. Cases were divided into retrospective, CES (clinical-exome sequencing) (*n* = 79), using CES after the previous preliminary analysis, and prospective (*n* = 18), CES as first-tier approach. The 34 retrospective cases with inconclusive CES results were screened using WES (whole exome sequencing) plus 3 more cases directly sequenced by WES. **b** Molecular study results by clinical subgroup. **c** Inheritance mode distribution. The fully (*n* = 46) and incompletely (*n* = 6) characterized cases were classified according to the Mendelian inheritance mode of the causative gene and grouped into autosomal dominant (AD), autosomal recessive (AR), or X-linked (XL) trait. Non-Mendelian inheritances (mitochondrial and triallelism) were also considered, as well as the cases with co-occurrence of two different genetic causes. **d** Final classification of the SRD cohort after genetic testing. Cases that were reclassified into another clinical subgroup due to the identification of the causative gene are indicated

Of the 100 probands, 82 retrospective cases were screened in this study using new clinical and/or exome approaches. The workflow used is described in Supplementary Fig. S1. In retrospective cases analyzed by CES, the diagnostic yield was 25% (20/79) (Fig. 3a).

In 34 retrospective cases with inconclusive findings using small gene panels, WES were sequentially performed to analyze an extended 447-gene panel. In addition, three more cases were directly sequenced by WES. The overall diagnostic yield of WES analysis was 43% (16/37; 14 totally and two partially conclusive characterized cases) (Fig. 3a), representing the highest diagnostic yield for retrospective cases. Considering only the groups of well-recognizable syndromes, the diagnostic rate of WES increased to 75% (6/8) (Supplementary Table S5). After the first WES data analysis of the 37 cases, in the 26 cases that remained uncharacterized, a WES reanalysis was conducted using new bioinformatic approaches, including variant reannotation with the latest updated tools and hypothesis-free variant prioritization. This reanalysis led to a characterization rate of 19% (5/26), also showing a higher yield for cases with well-recognizable syndromes (50%; 2/4) (Supplementary Table S5). The specific reasons that led to the characterization of cases during reanalysis are detailed in Supplementary Table S6.

In addition, 18 prospective cases were analyzed by means of CES as a first-tier approach, including SNVs and read-depth CNV analysis, with a significant diagnostic rate of 83% (15/18) (Fig. 3a and Supplementary Table S5).

For disease groups, the molecular diagnostic rate is described in Fig. 3b, in which 62% (26/42) of probands suspected to present well-recognizable syndromes (CILIOPATHY and SPECIFIC) were characterized, whereas, for the remaining groups of patients categorized according to the major extra-ocular symptoms, characterization decreased to 45% (26/58). Patients with an a priori suspicion of “CILIOPATHY” have been the most genetically diagnosed, with a detection rate of 66% (23/35).

The overall diagnostic yield in our cohort was 52%. Cases were classified according to their genetic outcome in four different subgroups (Supplementary Fig. S2). First, characterized cases, i.e. those with a fully conclusive molecular diagnosis, including 46 probands with likely causative variants that fully explained the phenotypic presentation and six cases presented likely causative variants that only explained part of the phenotype, but not the other concomitant symptoms. Among the uncharacterized cases, we identified six cases with monoallelic pathogenic variants in a recessive gene that fitted the phenotypic presentation and four probands with inconclusive molecular diagnosis due to the identification of a monoallelic VUS in a plausible gene to explain the phenotype presented. Finally, we did not identify any likely causal or pathogenic variants in 38 probands to date, even after the last data reanalysis performed in 2020 (Supplementary Table S5 and S6).

Segregation analyses were conducted in 55% of the families (34/62) in which at least one variant was found.

Most of the cases were isolated cases (77%) (Supplementary Table S8). The characterization rate between the familial and sporadic cases showed a trend to be significant (*p* = 0.0544).

### Mutational spectrum

A total of 75 VUS/likely pathogenic SNVs and 7 CNVs were found in 47 different genes (Supplementary Table S7 and Supplementary Fig. S3). Variants in 16 genes were identified in probands suspected of ciliopathy and three with specific rare diseases, and a case with suspicion of mtDNA disorder was characterized with a pathogenic variant in the mitochondrial *MT-ATP6* gene (MIM *516060). In the remaining four groups, variants in 31 different genes were found.

Among genes in which causative variants have been found, nine have been related to both autosomal dominant and recessive patterns of inheritance. Biallelic variants associated to recessive inheritance were found in five of those genes (*ACO2, PDE6B, PEX6, RDH12*, and *WFS1*) and were confirmed by parental segregation analysis in the 83% of them (five out of six cases). Moreover, four cases showed a monoallelic variant in one of those nine genes (*GLI1, HK1, OTX2* and *RHO*), including one de novo variant (RP-2176) (Supplementary Table S8). In all the cases with autosomal dominant inheritance suspicion, the presence of a second mutated allele (SNV or CNV) in the same gene was excluded and allele frequency and type of mutations were compatible with an autosomal dominant inheritance.

The five most mutated genes were *BBS1* (MIM *209901), *AHI1* (MIM *608894), *MKKS* (MIM *604896), *C8orf37* (MIM *614477), and *VPS13B* (MIM *607817). Moreover, the most prevalent allele in our cohort was the previously described p.Met390Arg in *BBS1* (NM_024649.4), with an allelic frequency of 6% (7/113 identified alleles) in four characterized probands (RP-2634, RP-2846, RP-2996, and RP-3115), occurring in homozygosis 3 times, and once in compound heterozygosis in a case with suspected triallelism, appearing together with a monoallelic variant in *BBS5* (MIM *603650).

The most prevalent mode of inheritance was autosomal recessive (79%), followed by autosomal dominant (9%), X-linked (2%), and mitochondrial (2%) (Fig. 3c). In addition, atypical inheritance presentations were found in 4 families. Interestingly, two suspected triallelic cases (4%) were found in two families (RP-2634 and RP-2966), involving ciliopathy-related genes. We identified a proband (RP-2634) carrying biallelic pathogenic variants (p.Met390Arg and c.951+58C>T) in *BBS1* with a monoallelic *BBS5* variant (p.Arg207His). This last variant did not appear in her affected sibling, who presented a milder non-syndromic phenotype. We additionally found a patient (RP-2966) to be homozygous for a novel splicing variant (c.156-1G>T) in *C8orf37* in combination with a novel monoallelic nonsense variant (p.Cys344*) in *WDPCP* (MIM *613580). In two probands (RP-1018 and RP-1321), a confirmed dual genetic diagnosis encompassing a genomic rearrangement and a monogenic disease was present (4%). First, in an affected girl (RP-1018), the presence of a ~ 4.75 Mb de novo deletion in chromosome X was observed within Xq22.32-22.2 (hg19: chrX:5748782-10477366), involving two OMIM genes, i.e., *NLGN4* and *MID1*, which are associated with Mental retardation, X-linked (MIM #300495) and Opitz GBBB syndrome, type I (MIM #300000), respectively. Although subsequent studies in peripheral blood could not demonstrate skewed inactivation of the X chromosome, this deletion, together with the presence of biallelic *USH2A* (MIM *608400) variants, may explain the phenotype of the patient suffering from early onset retinitis pigmentosa (RP), global developmental delay, tip-toe gait, talipes equinovarus, and dysmorphic features (Supplementary Fig. S4). The proband RP-1321, who presented RP, cochlear malformation, neurodevelopmental alterations, recurrent pneumonia, hypotonia, and limb malformations and carried a likely pathogenic hemizygous missense variant in *NYX* (MIM *300278) and a 1q21.1q21.2 microduplication of 1.2 Mb (Supplementary Fig. S5). This maternally inherited CNV produces a recognized autosomal dominant duplication syndrome with incomplete penetrance and variable expressivity (MIM #612475) of psychiatric/developmental disorders, articulation abnormalities, and hypotonia.

### Final clinical and genetic classification

A total of 46 SRD probands were fully genetically and phenotypically characterized (Supplementary Table S8) following the molecular studies. Those included two previously unclassified SRD probands (RP-1007 and RP-2879), now categorized as BBS and COH. Considering only those patients with a priori diagnosis, 27% (12/44) of cases were reclassified into another clinical subgroup after NGS-based genetic testing thanks to the identification of a clearly causative gene that explained the full phenotype (Supplementary Table S8).

Remarkably, we observed several likely new or extremely rare phenotypic associations for 8 genes in 17% (8/46) of the fully characterized probands (Table 1).

**Table 1:** New and rare phenotype–genotype associations among completely and partially characterized cases in the cohort

Case	Initial diagnosis (clinical category)	Gene (variants)	Associated phenotypes (OMIM)	New/rare association	Post-test diagnosis (clinical category)	Observations	PMID
RP-0094	RP + OPA + cataracts + HL (RD + HL)	***ACO2*** (p.Cys592Tyr and p.Arg767Cys)	OPA; ICRD	Hearing impairment (HP: 0000365)	RP + OPA + cataracts + HL (RD + HL)	Gene associated with a wide range of phenotypes, though deafness is highly uncommon	32449285; 32519519
RP-2310	RP + Asperger syndrome (RD + ND)	***ARL13B*** (c.57_59+16dup, homozygosis)	JBTS	Asperger syndrome	RP + ND (RD + ND)	Selective loss of ARL13B reported as one of the mechanisms underlying ASD	28787594
RP-2995	ALMS-like (CILIOPATHY)	***C8orf37*** (p.Asp10Lysfs*12, homozygosis)	RP; CRD; BBS	Sensorineural hearing impairment (HP: 0000407)	Atypical BBS (CILIOPATHY)	New association, since hearing complications are usually produced by chronic otitis media	22713813
RP-1436	SLSN-like (CILIOPATHY)	***IFT140*** (p.Gly1229Val and c.3661-9T>G)	RP; SRTD with/without polydactyly	Absence of skeletal findings	Early-onset RP + PKD (CILIOPATHY)	Patient presented a mild phenotype	–
RP-0132	RP + HL + DI + PD + SD (RD + SD)	***IFT81*** (p.Gln657*, homozygosis)	SRTD with/without polydactyly	Visual features together with skeletal findings	Skeletal ciliopathy (CILIOPATHY)	Visual and skeletal findings have been independently reported in association with *IFT81*, but not both kind of symptoms in the same patient	26275418; 32233951; 32783357
RP-2032	RP + CD + HL + ID (RD + HL ± ND)	***MCOLN1*** (c.878-1G>A, homozygosis)	ML	Hearing impairment (HP: 0000365)	Atypical ML (SPECIFIC)	No previous reports of a relationship between HL and *MCOLN1*	–
RP-1691	LCA + CM (RD + OTHER)	***OTX2*** (p.Ser169*)	MCOPS; CPHD	Atresia of the external auditory canal (HP: 0000413); microtia, third degree (HP: 0011267)	LCA + CM (RD + OTHER)	Absence of the characteristic neuroendocrine alterations or ocular developmental anomalies of the *OTX2*-related disorders	20486942; 29588463
RP-3018	RP + HL (RD + HL)	***PEX6*** (p.Glu439Glyfs*3 and p.Arg876Trp)	HMLR; PBD	Mild phenotype	RP + HL (RD + HL)	Pediatric patient; mandatory clinical reassessment in a few years to monitor the possible development of new symptoms commonly associated with *PEX6*-related disorders, as previously described	32214787

Reference transcripts: NM_001098.3 for *ACO2*, NM_001174150.1 for *ARL13B*, NM_177965.3 for *C8orf37*, NM_014714.4 for *IFT140*, NM_001143779.2 for *IFT81*, NM_020533.3 for *MCOLN1*, NM_001270525.1 for *OTX2*, and NM_000287.4 for *PEX6*

*ASD* autism spectrum disorders, *BBS* Bardet–Biedl syndrome, *CD* corneal dystrophy, *CM* congenital malformations, *CPHD* pituitary hormone deficiency, combined, *CRD* cone-rod dystrophy, *DFNB* deafness, *DI* diabetes insipidus, *HL* hearing loss, *HMLR* Heimler syndrome, *ICRD* infantile cerebellar-retinal degeneration, *ID* intellectual disability, *JBTS* Joubert syndrome, *MCOPS* microphthalmia, syndromic, *ML* Mucolipidosis, *ND* neurodevelopmental disorder, *OPA* optic atrophy, *PBD* Peroxisome Biogenesis Disorder, *PD* psychiatric disorder, *PKD* polycystic kidney disease, *RP* retinitis pigmentosa, *SRTD* short-rib thoracic dysplasia

Regarding disease-groups reclassification, characterized probands suspected of well-recognizable syndromes (CILIOPATHY and SPECIFIC) were reclassified less frequently (16%; 4/25) after the molecular studies than the characterized patients grouped according to their non-specific extra-ocular symptoms (42%; 8/19). Interestingly, we obtained a higher clinical accuracy in the group of ciliopathies (82%; 18/22). Reclassification rates above 33% were achieved in the clinical subgroup with RD + HL and/or neurodevelopmental alteration (Fig. 3d).

**Fig. 4:**
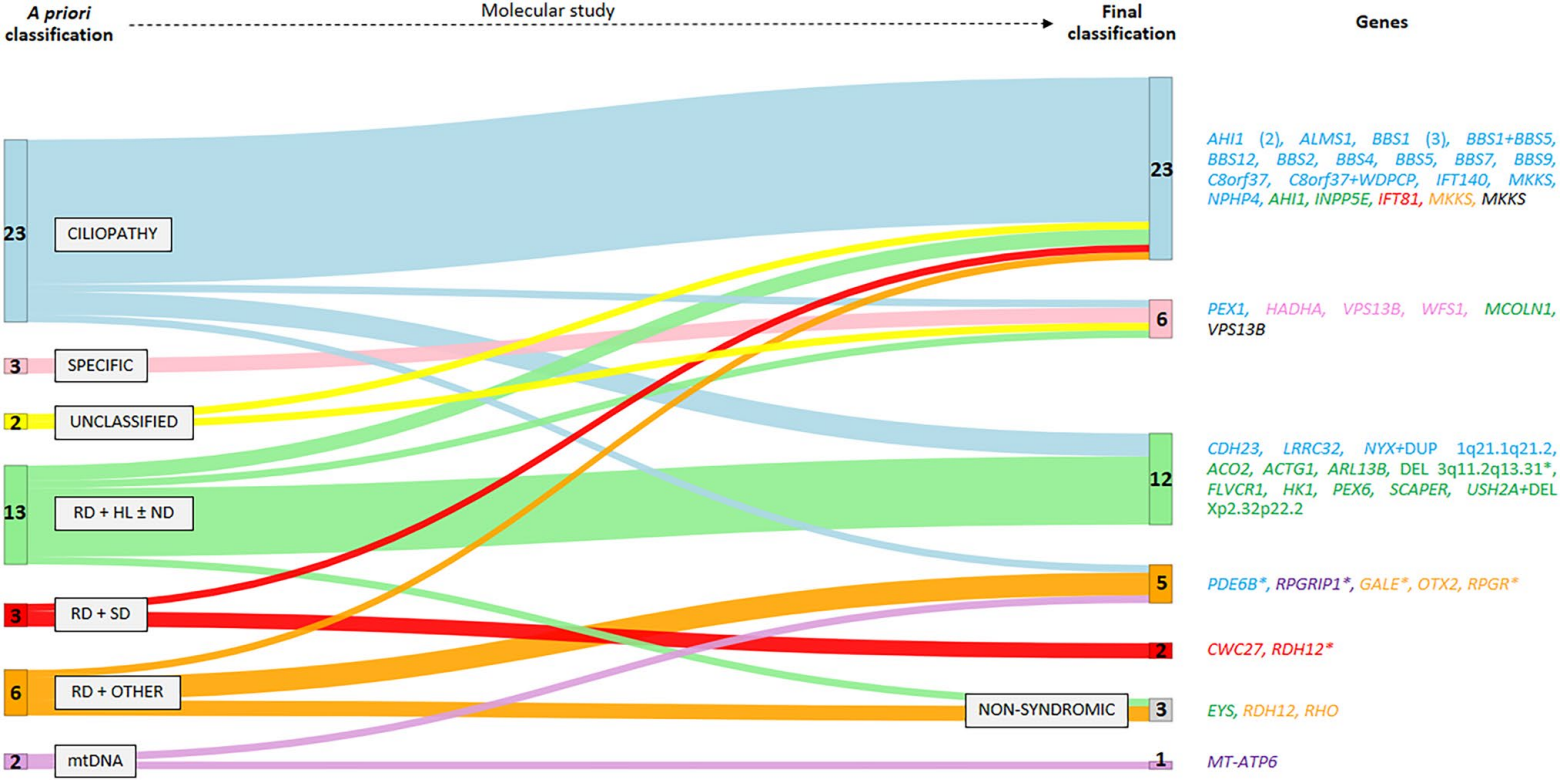
Sankey chart of the clinical distribution of the characterized cases before and after molecular studies and the results of this analysis. The causative genes appear with the color corresponding to the clinical group to which each case was assigned a priori. Sankey chart was created using the Sankey Diagram Generator by Dénes Csala, based on the Sankey plugin for D3 by Mike Bostock (https://sankey.csaladen.es/; 2014). *Phenotypically partially characterized. *DEL* deletion, *DUP* duplication, *RD* retinal dystrophy, *HL* hearing loss, *mtDNA* mitochondrial DNA, *ND* neurodevelopmental disorder, *SD* skeletal disorder

As detailed in Fig. 4, three cases without a priori suspicion of well-known SRD were found to be carrying likely causal variants in non-syndromic RD-associated genes. In the proband RP-1022, who presented RP, optic neuropathy, and mild unilateral hearing impairment, the homozygous missense variant p.(Ser2983Tyr) in *EYS* (MIM *612424) was found; therefore, this case was reclassified as non-syndromic. Patient RP-0118 had early-onset RP and type 1 diabetes mellitus and carried the heterozygous variant p.(Arg132Trp) in *RHO* (MIM *300023), the most frequent gene in autosomal dominant RP; thus, he was reclassified to non-syndromic. Finally, the proband RP-1175 with early-onset RP and obesity was solved with a homozygous missense variant p.(Leu93Pro) in *RDH12* (MIM *608830) associated with non-syndromic RP. In those three cases, the isolated extra-ocular symptom does not seem to be associated with the genetic cause of the retinal presentation and is likely explained by a non-genetic cause.

After genetic testing, six cases were partially characterized as not completely fitting the expected phenotype linked to the causative gene (Supplementary Table S8). In four cases (RP-0567, RP-1021, RP-1702, and RP-3047), visual alteration was clearly explained by causal variants in early-onset RD genes, i.e., *RPGRIP1* (MIM *605446)*, PDE6B* (MIM *180072)*, RPGR* (MIM *312610), and *RDH12*. However, other systemic presentations, including myopathic alterations, glomerulonephritis, preaxial polydactyly, genital abnormalities, and dysmorphic features, remained unsolved. On the other hand, the visual phenotype was apparently not related to the genetic findings in two families. In two affected siblings belonging to family RP-0897, we identified two compound heterozygous variants in *GALE*. This recessive gene is associated with mild galactose deficiency (MIM #230350), which seems to explain the fact that the family only presented cognitive delay, hepatomegaly, and splenomegaly. However, GALE deficiency has never been associated with retinal dystrophies (RD). In the proband RP-3055 suffering from a complex presentation of syndromic cone-rod dystrophy, we identified a heterozygous microdeletion of ~ 16.5 Mb on 3q11.2q13.31 (hg19: chr3:97483365-113953480), involving at least 60 OMIM genes, which included *ARL6* (Bardet-Biedl syndrome, MIM #600151)*, IMPG2* (retinitis pigmentosa, MIM #613581; and macular dystrophy, vitelliform, MIM #616152)*, ZBTB11* (Intellectual developmental disorder, MIM #618383), and *ATP6V1A* (developmental and epileptic encephalopathy, MIM #618012), among others*.* This microdeletion has been associated with chromosome 3q13.31 deletion syndrome (MIM #615433), thus, it could explain the cognitive impairment of our patient. However, visual findings and other extraocular alterations remain unexplained.

Another six probands presented a monoallelic pathogenic variant in a gene that could fully or partially explain the phenotype. On the one hand, we were able to obtain a partial genetic characterization related with their full (RP-1483 and RP-2001) or partial (RP-0620 and RP-1854) a priori diagnosis in four of them. In addition, on the other two probands carrying previously reported monoallelic *USH2A* variant (RP-0838) and monoallelic *BBS1* and *BBS5* variants (RP-1581) (Sanchez-Navarro et al. 2018), no additional findings were obtained following the reanalysis of NGS data.

Additionally, we found four carriers of monoallelic VUS in a gene related with their phenotype (RP-0249, RP-2184, RP-2394, and RP-2882) (Supplementary Table S8).

## Discussion

SRDs are a highly heterogeneous group of ocular diseases characterized by a challenging molecular characterization and clinical management (Shaheen et al. 2016; Tatour and Ben-Yosef 2020). In the literature, it is rather frequent to find studies focused on specific syndromes (Knopp et al. 2015; Vilboux et al. 2017) or single panel-based studies (Shaheen et al. 2016; Jiman et al. 2020), which provide a biased landscape of SRDs. We present the study of a cohort comprising naïve and previously studied cases of well-known syndromes and unspecific SRD patients from a single center, focusing on the challenges derived from diagnosis and molecular characterization.

Within the ~ 80 SRDs already described, the most common subgroup is ciliopathies (Tatour and Ben-Yosef 2020), BBS being the most frequent condition representing 36% of our cohort (Perea-Romero et al. 2021); this result is similar to the 25–45% frequencies related to non-Usher syndromic families reported in different populations around the world (Motta et al. 2018; Sharon et al. 2020; Holtan et al. 2020).

Apart from well-defined syndromes, SRDs can appear as the combination of visual alterations along with one or more affected systems, where the most common are those involving the CNS (68%) and the ear (33%) (Yang et al. 2020; Tatour and Ben-Yosef 2020). These data are corroborated by the case distribution in our cohort, with rates of 59% and 28%, respectively.

The overall characterization rate achieved in our study was 52% using mainly targeted gene panel testing but also WES analysis; this rate is similar to that observed in other cohorts (Manara et al. 2019; Jiman et al. 2020), ranging between 55% and 60% (Supplementary Table S9). However, a diagnostic rate of 85% was reported in a cohort formed exclusively by consanguineous families with ciliopathies (Shaheen et al. 2016). A similar yield was observed in our prospective analysis of 18 consecutive cases referred over a 3-year period using clinical-exome sequencing to perform a simultaneous analysis of SNVs and CNVs. Molecular diagnostic rates for SRD are highly variable, ranging from 25 to 66% for specific phenotypic classification. Higher yields were obtained for patients presenting well-recognized syndromes, even when smaller gene panels were used in CES. This highlighted the importance of deep phenotyping in delineating SRDs.

However, the high rates obtained in other patients with less specific systemic presentations clearly indicate the utility of our approach to identify the underlying genetic causes and for differential diagnosis. This is the case for patients with retinal disorders accompanied with an isolated extra-ocular symptom. In 27% of patients within this category, RD is finally explained by a non-syndromic gene. Moreover, half of the a priori unclassified cases were successfully grouped after molecular testing. Thus, this NGS-based approach helps to improve the prognostic and management of the patients.

WES has been established as the best approach for the study of SRD cases, as it improves molecular diagnosis and understanding due to its efficacy in identifying new variants, phenotype-genotype correlations, and causative genes (Vilboux et al. 2017; Gupta et al. 2017; Boczek et al. 2018; Abu Diab et al. 2019; Jaffal et al. 2019; Cogné et al. 2020).

Several studies described the importance of periodic NGS reanalysis to increase diagnostic rates up to 30% in unsolved patients (Wenger et al. 2017; Wright et al. 2018b; Alfares et al. 2018; Ewans et al. 2018; Jalkh et al. 2019; Li et al. 2019; Liu et al. 2019) depending on the spectrum of genetic disorders analyzed, type of NGS, mode of reanalysis, or time period since the first analysis (Supplementary Table S10). Specifically, the WES reanalysis may allow characterization of an additional 30% of patients with WES without the need for whole-genome sequencing (WGS) (Alfares et al. 2018). In our cohort, the WES-related reanalysis yield is 19% due to the application of a hypothesis-free approach in genomic data analysis, the description of a new gene (without known association on first analysis), and the compilation of new data from clinical reassessment of the patient.

According to our data and clinical routine, we suggest carrying out reanalysis of the data from RD cases previously studied by NGS in these circumstances: (1) cases with new clinical or family information; and (2) cases in which a new candidate gene related to clinical presentation is reported in the literature. However, if in the 2 years following first analysis, these circumstances are not met, a reanalysis of NGS data should be undertaken in the light of the large volume of newly published information.

One of the problems related to the use of NGS using large gene panels or hypothesis-free approaches is the identification of VUS and the underlying difficulties associated to interpretation, since they are frequently the only positive finding in molecular testing (Frebourg 2014). In our cohort, 4% of cases were carriers of monoallelic VUS in a recessive gene related with their phenotypic presentation. Similarly, another six patients showed monoallelic class 4/5 variants in recessive genes found in this or previous studies (Sanchez-Navarro et al. 2018). To solve those cases, it is highly recommended to periodically reassess the clinical significance of the VUS and also carry out further WGS, in order to search for a potential second pathogenic allele in untargeted regions such as deep-intronic or regulatory elements.

Ninety-eight percent of the unsolved cases in this cohort underwent targeted panel testing, and WES analysis was not carried out in only 42% of them. In all these patients, particularly in unsolved WES patients, WGS will be the best option to identify potential novel genes/variants or non-coding mutations that remain to be determined.

To date, many reports have highlighted the existence of co-occurrence of IRD with a rare non-ocular phenotype in SRD patients, this being an important matter in clinical management and follow-up (Ehrenberg et al. 2019). The incidence of a proven dual genetic diagnosis has been established in the range of 4.6% and 4.9% in previous reports (Yang et al. 2014; Posey et al. 2017), similar to our 4.3% value. In our cohort, dual genetic diagnosis was obtained by the combination of genomic rearrangement and a monogenic disease produced by a non-syndromic IRD gene (RP-1018 and RP-1321); this finding is in line with a previously reported case by our group of a proband solved with biallelic variants in *USH2A* (USH) and a de novo microdeletion at 17q21.31 (Koolen-de Vries syndrome, MIM #610443) (Sanchez-Navarro et al. 2018). Moreover, our dual genetic diagnostic rate could be underestimated owing to the fact that there are six probands with partial diagnosis (RP-0567, RP-0897, RP-1021, RP-1702, RP-3047, and RP-3055) that remain to be fully characterized due to the likelihood of an undiscovered second genetic disease. So, in partially characterized cases with genes associated with IRDs, genomic rearrangements might be studied, while in probands, in which only SRD genes have been analyzed, both IRD genes and large CNVs should be screened.

As new research evidence is published, new genotype–phenotype associations are discovered in SRDs, delineating its phenotypic spectrum and improving molecular diagnosis, as well as revealing the value of a robust previous clinical analysis (Abu Diab et al. 2019; Shamseldin et al. 2020). Moreover, in 8 of the characterized cases, some of the clinical features of the patient did not completely fit with the known syndrome related to the causative gene found. This may be either a fortuitous association or an expansion of the already known phenotype. For instance, a priori RP-2995 was suspected of ALMS-like, but after the molecular characterization, a homozygous frameshift variant in *C8orf37* was found. This therefore presents an atypical BBS with sensorineural hearing loss, a rare finding in this syndrome due to the fact that hearing complications are usually produced by chronic otitis media (Forsythe and Beales 2013). Sensorineural hearing impairment has been described once, in a genetically uncharacterized patient (Singh et al. 2017); our study, however, marks the first time that the reported patient has been molecularly characterized. Another example of a new association was found in the patient RP-2310, which was characterized with a homozygous splicing variant in *ARL13B* (MIM *608922). Normally, the gene *ARL13B* has been associated with JBTS and the ciliopathy spectrum; in this case, RP-2310 had rod-cone dystrophy and Asperger syndrome, which is part of the autism spectrum disorders (ASD). Selective loss of ciliary ARL13B in interneurons has been shown to alter their morphology and synaptic connectivity, leading to the disturbance of the excitatory/inhibitory excitatory balance, which is one of the mechanisms underlying neurodevelopmental disorders such as ASD (Guo et al. 2017).

In summary, this study reports findings on a heterogeneous SRD cohort and its genetic diagnostic management, expanding the phenotypical spectrum in these diseases and pointing to the importance of an extensive and accurate clinical analysis together with an adequate genetic study and choice of the NGS technology according to the specific characteristics of each case. We can conclude that HPO terms are a suitable tool for phenotypic description in SRD patients; however, based on our observations, subsequent application of established clinical criteria is highly recommendable. Furthermore, the use of well-designed and curated virtual panels leads to the best characterization rates, regardless of the NGS approach used (CES or WES). Bioinformatic analysis should include CNV evaluation and periodical reanalysis. This approach increases the diagnostic yield from 41 to 52%. Besides, it is important to note that the presence of some extra-ocular findings together with a visual disease is not always due to a single genetic cause. In our cohort there are SRD cases explained by (1) non-syndromic IRD with a non-genetic disease and (2) two different genetic diseases in the same patient. Moreover, the dual genetic diagnoses presented here were mainly due to the co-occurrence of non-syndromic IRD together with a genomic rearrangement. Thus, not only SRD genes should be considered, but also large CNVs should be screened in presumed SRD cases. Finally, inclusion of non-syndromic IRD genes in the study of SRD cases may help to uncover new phenotypic associations.

## Supplementary Information

Below is the link to the electronic supplementary material.

Supplementary file1 (PDF 601 kb)

Supplementary file2 (XLSX 77 kb)

## Data Availability

NGS data are available in public, open access repositories such as the European Genome-Phenome Archive (EGA; https://www.ebi.ac.uk/ega/home; EGAD00001005746, EGAD00001005498 and EGAD00001007022), RD-Connect (https://rd-connect.eu/) and the Collaborative Spanish Variant Server (CSVS; http://csvs.babelomics.org/) as aggregated data. The rest of the data are available upon reasonable request.

## Code availability

An updated version of the bioinformatic tools of pipeline 1 and 2 is available online at https://github.com/TBLabFJD/VariantCallingFJD.
