# Supplementary material for: NGS and phenotypic ontology-based approaches increase the diagnostic yield in syndromic retinal diseases
Source: Hum Genet. 2021 Aug 26;140(12):1665–78. doi: 10.1007/s00439-021-02343-7 (PMC8553673; doi:10.1007/s00439-021-02343-7)
Supplement: Supplementary file 1 — Supplementary file1 (PDF 601 kb) [file 439_2021_2343_MOESM1_ESM.pdf]

NGS and phenotypic ontology-based approaches increase the diagnostic yield in syndromic retinal diseases

Perea-Romero I[1,2], Blanco-Kelly F[1,2], Sanchez-Navarro I[1], Lorda-Sanchez I[1,2], Tahsin-Swafiri S[1,2], Avila-Fernandez A[1,2], Martin-Merida I[1,2], Trujillo-Tiebas MJ[1,2], Lopez-Rodriguez R[1,2], Rodriguez de Alba M[1], Iancu IF[1,2], Romero R[1,2], Quinodoz M[3,4,5], Hakonarson H[6,7,8], Minguez P[1,2], Corton M[1,2], Rivolta C[3,4,5], Ayuso C[1,2]

[1] Health Research Institute-Fundación Jiménez Díaz University Hospital, Universidad Autónoma de Madrid (IIS-FJD, UAM), Madrid, Spain.

[2] Center for Biomedical Network Research on Rare Diseases (CIBERER), Instituto de Salud Carlos III, Madrid, Spain.

[3] Institute of Molecular and Clinical Ophthalmology Basel (IOB), Basel, Switzerland.

[4] Department of Ophthalmology, University of Basel, Basel, Switzerland.

[5] Department of Genetics and Genome Biology, University of Leicester, Leicester, UK.

[6] Center for Applied Genomics, Children's Hospital of Philadelphia, Philadelphia, PA, USA.

[7] Division of Human Genetics, Children's Hospital of Philadelphia, Philadelphia, PA, USA.

[8] Department of Pediatrics, Perelman School of Medicine, University of Pennsylvania, Philadelphia, PA, USA.

ONLINE RESOURCE 1

Supplementary Table S1 Clinical classification of patients with syndromic retinal diseases. Probands were classified in 7 categories. For patients with a well-recognizable SRD (i and ii), major, minor and supportive signs associated to each clinical entity and diagnostic criteria used to classify cases are indicated. The remainder ungrouped cases were classified into 5 additional categories (iii to vii), using a classification based on Human Phenotype Ontology (HPO) terms for the main extra-ocular symptoms. In a patient, the main HPO terms may be accompanied by any other clinical feature. Underlined terms corresponded to the more general parent term

Category	Disease	Major criteria (MC)	Minor criteria (mc)	Supportive evidence (SE)	Disease diagnostic criteria	Disease-like diagnostic criteria
**i) Ciliopathy (CILIOPATHY)**	**ALMS** (Marshall et al. 2007)	- Vision problems: nystagmus, cone/cone-rod dystrophy, rod-cone dystrophy, blindness	- Obesity, type II diabetes mellitus and/or insulin resistance<br>- Sensorineural hearing impairment<br>- Dilated cardiomyopathy<br>- Decreased liver function<br>- Renal insufficiency<br>- Male hypogonadism. Females: menstrual irregularities and/or abnormal circulating androgen level<br>- Short stature	- Global developmental delay<br>- Hypothyroidism<br>- Growth hormone deficiency<br>- Hyperlipidemia<br>- Scoliosis<br>- *Pes planus*<br>- Normal digits[a]<br>- Alopecia<br>- Hypertension<br>- Recurrent pneumonia<br>- Recurrent urinary tract infections, abnormality of the urinary system	a) 2 MC and 2 mc<br>or<br>b) 1 MC and 4 mc	1 MC, 2 mc and 2 SE
	**BBS** (Forsythe and Beales 2013)	- Rod-cone dystrophy<br>- Postaxial polydactyly<br>- Obesity<br>- Abnormality of the female and male genitalia<br>- Abnormality of the kidney<br>- Specific learning disability, intellectual disability	- Delayed speech and language development<br>- Global developmental delay<br>- Type II diabetes mellitus<br>- Abnormality of the teeth<br>- Abnormal heart morphology<br>- Brachydactyly syndrome, syndactyly<br>- Ataxia, poor coordination<br>- Anosmia, hyposmia	- Chronic otitis media<br>- Aganglionic megacolon (Hirschprung disease)	a) 4 MC<br>or<br>b) 3 MC and 2 mc	a) At least, 3 MC and 1 mc<br>or<br>b) less MC, but including postaxial polydactyly
	**JBTS** (Parisi et al. 2007)	- Molar tooth sign on MRI	- Abnormal eye movements: oculomotor apraxia, nystagmus	- Scoliosis<br>- Seizures	3 MC	a) 2 MC and 2 mc<br>or

		- Infantile muscular hypotonia<br>- Intellectual disability, global developmental delay	- Irregular respiration<br>- Encephalocele<br>- Polymicrogyria<br>- Other CNS findings: agenesis of corpus callosum, heterotopia, Dandy-Walker malformation, cortical dysplasia<br>- Vision problems: retinal dystrophy, congenital blindness<br>- Polycystic kidney dysplasia<br>- Nephronophthisis<br>- Polydactyly	- Ataxia		b) 1 MC and 3 mc
	**SLSN** (Parisi et al. 2007)	- Retinal dystrophy<br>- Nephronophthisis	- Molar tooth sign on MRI<br>- Muscular hypotonia<br>- Intellectual disability, global developmental delay<br>- Abnormal eye movements: oculomotor apraxia, nystagmus<br>- Encephalocele<br>- Hepatic fibrosis	N/A	3 MC	Retinal dystrophy and any renal abnormality
**ii) Non-ciliary specific SRD (SPECIFIC)**	**ATS** (Kashtan 1993; Nozu et al. 2019)	- Renal involvement: hematuria, proteinuria, chronic kidney disease	- Bilateral sensorineural hearing impairment<br>- Ocular abnormalities: anterior lenticonus, retinal flecks, posterior subcapsular cataract<br>- Diffuse leiomyomatosis	- Aortic dilatation	1 MC and 2 mc	1 MC and 1 mc
	**CLN** (Kohlschütter et al. 2019)	- Seizures, epilepsy<br>- Dementia<br>- Retinopathy<br>- Motor function impairment: involuntary movements, myoclonus, ataxia, spasticity<br>- Morphological abnormality of the CNS:	N/A	N/A	4 MC	At least, 2 MC

	accumulation of autofluorescent lipopigment				
**COH** (Wang et al. 1993)	- Retinal dystrophy (childhood), severe myopia (progressive)<br>- Microcephaly<br>- Intellectual disability, global developmental delay<br>- Joint hypermobility<br>- Childhood-onset truncal obesity<br>- Abnormal social behaviour<br>- Neutropenia<br>- Typical COH syndrome facial gestalt: thick hair and eyebrows, long eyelashes, downslanted palpebral fissures, broad nasal tip, smooth and short philtrum, facial hypotonia	- Muscular hypotonia<br>- Short stature<br>- Small and narrow hand and foot	- Growth hormone deficiency<br>- Chronic otitis media<br>- High-pitched and weak cry<br>- Oral ulcers, gingival overgrowth<br>- Oral ulcers, gingival overgrowth<br>- Musculoskeletal features: kyphosis, scoliosis, pes planus	6 MC	4 MC and 1 mc
**LCHAD deficiency** (den Boer et al. 2002)	- Laboratory findings: long chain 3 hydroxyacyl CoA dehydrogenase deficiency	- Cardiomyopathy<br>- Hepatomegaly<br>- Hypoglycemia<br>- Muscular hypotonia<br>- Pigmentary retinopathy	N/A	Laboratory findings	N/A
**MUL** (Karlberg et al. 2004)	- Intrauterine growth retardation, postnatal growth retardation or short stature<br>- Radiological findings: thickened cortex of long bones/slender long bones with narrow diaphyses or J-shaped *sella turcica*	- High pitched voice<br>- Hepatomegaly<br>- Nevus<br>- Fibrous dysplasia of long bone[a]	- Ventriculomegaly<br>- Dysarthria<br>- Muscular hypotonia<br>- Abnormal heart morphology	a) 3 MC and 1 mc<br>or<br>b) 2 MC and 3 mc	3 MC and 4 SE

		- Ocular findings: pigmentary retinopathy<br>- Craniofacial features: dolichocephaly, triangular face, high and broad forehead and *telecanthus*				
	**WFS** (Tranebjærg et al. 1993)	- Diabetes mellitus (juvenile-onset)<br>- Optic atrophy (juvenile-onset)	- High-frequency sensorineural hearing impairment<br>- Truncal or gait ataxia<br>- Dementia, intellectual disability<br>- Psychiatric disease<br>- Neurogenic bladder<br>- Cardiomyopathy, abnormal heart morphology<br>- Endocrine alterations: central diabetes insipidus, hypothyroidism, growth delay, delayed puberty, hypogonadism (in males)	- Other ophthalmologic findings: cataract, pigmentary retinopathy, nystagmus<br>- Apnoea<br>- Gastrointestinal dysmotility	2 MC and, at least, 1 mc (normally, hearing impairment)	a) 2 MC<br>or<br>b) 1 MC and 1 mc or additional SE

**Category**	**Main HPO terms**
**iii) RD + HL and/or ND (RD+HL±ND)**	Hearing impairment (HP: 0000365)<br>Neurodevelopmental abnormality (HP: 0012759): neurodevelopmental delay (HP: 0012758)<br>Abnormality of nervous system physiology (HP: 0012638): abnormality of higher mental function (HP: 0011446), morphological abnormality of the central nervous system (HP: 0002011), abnormality of movement (HP: 0100022), ataxia (HP: 0001251), seizures (HP: 0001250)
**iv) Neuropathy, myopathy, or suspicion of mtDNA disorder (mtDNA)**	Abnormality of metabolism/homeostasis (HP: 0001939): abnormality of carbohydrate metabolism/homeostasis (HP: 0011013), abnormality of lipid metabolism (HP: 0003119), abnormality of nitrogen compound homeostasis (HP: 0004364), abnormality of the mitochondrion (HP: 0012103)<br>Abnormality of the musculature (HP: 0003011): abnormality of muscle physiology (HP: 0011804), abnormal muscle tone (HP: 0003808)<br>Abnormality of coordination (HP: 0011443): poor motor coordination (HP: 0002275), incoordination (HP: 0002311)
**v) RD + SD (RD+SD)**	Abnormality of skeletal morphology (HP: 0011842): abnormal bone structure (HP: 0003330), abnormal axial skeleton morphology (HP: 0009121), abnormal appendicular skeleton morphology (HP: 0011844), preaxial polydactyly (HP: 0100258), abnormal joint morphology (HP: 0001367)

	Growth delay (HP: 0001510)
**vi) RD + other (RD+OTHER)**	Abnormality of the endocrine system (HP: 0000818): type I diabetes mellitus (HP: 0100651)<br>Obesity (HP: 0001513)<br>Abnormality of the cardiovascular system (HP: 0001626): abnormal heart morphology (HP: 0001627), abnormality of the vasculature (HP: 0002597)<br>Abnormal genital system morphology (HP: 0012243)<br>Abnormality of blood and blood-forming tissues (HP: 0001871)<br>Other terms not included in the rest of categories
**vii) Unclassified due to the lack of clinical information (UNCLASS/UNCLASSIFIED)**	N/A

ALMS, Alström syndrome; ATS, Alport syndrome; BBS, Bardet-Bield syndrome; CLN: ceroid lipofuscinosis, neuronal; CNS, central nervous system; COH, Cohen syndrome; HL: hearing loss; JBTS, Joubert syndrome; LCHAD deficiency: long-chain 3-hydroxiacyl-CoA dehydrogenase deficiency; MRI, magnetic resonance imaging; mtDNA: mitochondrial DNA; MUL: Mulibrey nanism; N/A: non-available; ND: neurodevelopmental disorder; RD: retinal dystrophy; SD: skeletal disorder; SLSN, Senior-Løken syndrome; WFS, Wolfram syndrome. [a] Clinical entity without HPO term

Supplementary Table S2 Summary of the list of genes included on each analysis

Name	Technique	Genes included
136-gene virtual panel	CES	*ABCC6, ABHD12, ACBD5, ADAMTS18, ADGRV1, AHI1, ALMS1, ARL13B, ARL3, ARL6, ATXN7, B9D1, B9D2, BBIP1, BBS1, BBS10, BBS12, BBS2, BBS4, BBS5, BBS7, BBS9, C2ORF71, C5ORF42, C8orf37, CC2D2A, CDH23, CENPF, CEP164, CEP290, CEP41, CIB2, CLN3, CLN5, CLN6, CLN8, CLRN1, CNNM4, COL11A1, COL2A1, COL9A1, COL9A2, COL9A3, CSPP1, CTSD, CTSF, DNAJC5, DYNC2H1, FAM161A, FLVCR1, GLIS2, GNPTG, GRN, HARS, IFT140, IFT172, IFT27, IFT57, IFT80, IFT81, INPP5E, INVS, IQCB1, JAG1, KCNJ13, KIF11, KIF7, LCA5, LZTFL1, MAK, MFSD8, MKKS, MKS1, MTTP, MYO7A, NEK8, NOTCH2, NPHP1, NPHP3, NPHP4, OFD1, OTX2, PANK2, PCDH15, PCYT1A, PDE6D, PDZD7, PEX1, PEX2, PEX7, PGK1, PHYH, PLK4, POC1B, PPT1, PRPS1, RDH11, RP1, RP1L1, RP2, RPGR, RPGRIP1, RPGRIP1L, SDCCAG8, SLC41A1, SLC9A6, TCTN1, TCTN2, TCTN3, TMEM138, TMEM216, TMEM231, TMEM237, TMEM67, TOPORS, TPP1, TREX1, TRIM32, TRPM1, TTC21B, TTC8, TTPA, TUB, TULP1, USH1C, USH1G, USH2A, VCAN, VPS13B, WDPCP, WDR19, WDR34, WDR60, WFS1, XPNPEP3, ZNF423*
377-gene virtual panel	CES	*ABCA4, ABCB6, ABCC6, ABHD12, ACBD5, ACTB, ADAM9, ADAMTS10, ADAMTS17, ADAMTS18, ADAMTSL4, ADGRV1, ADIPOR1, AFG3L2, AGK, AHI1, AHR, AIPL1, ALDH3A2, ALMS1, ALX1, ANTXR1, AP3B1, ARL13B, ARL3, ARL6, ATF6, ATOH7, ATXN7, B3GLCT, B9D1, B9D2, BBS1, BBS10, BBS12, BBS2, BBS4, BBS5, BBS7, BBS9, BCOR, BEST1, BFSP1, BFSP2, BLOC1S6, BMP4, BMP7, C2orf71, C5orf42, C8orf37, CA4, CABP4, CACNA1F, CACNA2D4, CC2D2A, CDH23, CDH3, CDHR1, CDON, CEP164, CEP290, CEP41, CERKL, CHM, CHMP4B, CHRDL1, CIB2, CISD2, CLN3, CLN5, CLN6, CLN8, CLRN1, CNGA1, CNGA3, CNGB1, CNGB3, CNNM4, COL11A1, COL11A2, COL18A1, COL2A1, COL4A1, COL4A2, COL9A1, COL9A2, COL9A3, CRB1, CRX, CRYAA, CRYAB, CRYBA1, CRYBA4, CRYBB1, CRYBB2, CRYBB3, CRYGB, CRYGD, CRYGS, CTDP1, CTSD, CYP1B1, CYP27A1, CYP4V2, DHDDS, DNAJC5, DNM1L, DTNBP1, DYNC2H1, EDNRB, EFEMP1, ELOVL4, EPHA2, ERCC1, ERCC2, ERCC5, ERCC6, EYA1, EYS, FAM161A, FBN1, FGFR1, FLVCR1, FOXC1, FOXD3, FOXE3, FOXL2, FRAS1, FREM1, FREM2, FRMD7, FSCN2, FYCO1, FZD4, GCNT2, GDF3, GDF6, GJA3, GJA8, GLA, GLI2, GLIS2, GNAT1, GNAT2, GNB3, GNPTG, GPR143, GPR179, GRIP1, GRK1, GRM6, GRN, GUCA1A, GUCA1B, GUCY2D, HADHA, HARS, HCCS, HESX1, HGSNAT, HK1, HMCN1, HMX1, HPS1, HPS3, HPS4, HPS5, HPS6, HSF4, IDH3B, IFT140, IFT172, IFT80, IFT81, IMPDH1, IMPG2, INVS, IQCB1, ITM2B, JAG1, JAM3, KCNJ13, KCNV2, KIF11, KIF7, KLHL7, LAMA1, LCA5, LIM2, LMX1B, LRAT, LRIT3, LRP5, LTBP2, LYST, LZTFL1, MAF, MAK, MC1R, MERTK, MFN2, MFRP, MFSD8, MIP, MITF, MKKS, MKS1, MLPH, MTTP, MVK, MYH9, MYO5A, MYO7A, MYOC, NAA10, NBAS, NDP,*

		NEK8, NEUROD1, NHS, NMNAT1, NOTCH2, NPHP1, NPHP3, NPHP4, NR2E3, NR2F1, NRL, NYX, OAT, OCA2, OCRL, OFD1, OPA1, OPA3, OPN1SW, OTX2, P3H2, PANK2, PAX2, PAX3, PAX6, PCDH15, PDE6A, PDE6B, PDE6C, PDE6G, PDE6H, PDZD7, PEX1, PEX2, PEX6, PEX7, PGK1, PHYH, PIGL, PIK3R1, PITPNM3, PITX2, PITX3, PLA2G5, PNPLA6, POMGNT1, PORCN, PPT1, PRCD, PROKR2, PROM1, PRPF3, PRPF31, PRPF6, PRPF8, PRPH2, PRPS1, PRSS56, PXDN, RAB18, RAB27A, RAB3GAP1, RAB3GAP2, RAX, RAX2, RB1, RBP3, RBP4, RCBTB1, RD3, RDH12, RDH5, RGR, RGS9, RGS9BP, RHO, RIMS1, RLBP1, ROM1, RP1, RP1L1, RP2, RP9, RPE65, RPGR, RPGRIP1, RPGRIP1L, RS1, SAG, SALL4, SDCCAG8, SEMA3E, SEMA4A, SHH, SIL1, SIX3, SIX6, SLC16A12, SLC24A1, SLC24A5, SLC33A1, SLC41A1, SLC45A2, SLC9A6, SMOC1, SNAI2, SNRNP200, SNX3, SOX10, SOX2, SOX3, SPATA7, SPG7, STRA6, TBX22, TCTN1, TCTN2, TDRD7, TEAD1, TFAP2A, TGFB2, TIMM8A, TIMP3, TMEM114, TMEM126A, TMEM138, TMEM216, TMEM237, TMEM67, TOPORS, TPP1, TREX1, TRIM32, TRPM1, TSFM, TSPAN12, TTC21B, TTC8, TTPA, TUB, TUBGCP6, TULP1, TYR, TYRP1, UNC119, USH1C, USH1G, USH2A, VAX1, VCAN, VIM, VPS13B, VSX2, WDPCP, WDR19, WFS1,WHRN, XPNPEP3, ZNF423, ZNF513
447-gene virtual panel	WES	*ABCA4, ABCB6, ABCC6, ABHD12, ACBD5, ACO2, ACTB, ACTG1, ADAM9, ADAMTS10, ADAMTS17, ADAMTS18, ADAMTSL4, ADGRV1, ADIPOR1, AFG3L2, AGK, AHI1, AHR, AIPL1, ALDH3A2, ALMS1, ALPK1, ALX1, ANTXR1, AP3B1, ARL13B, ARL3, ARL6, ARMC9, ARSG, ATF6, ATOH7, ATXN7, B3GLCT, B9D1, B9D2, BBIP1, BBS1, BBS10, BBS12, BBS2, BBS4, BBS5, BBS7, BBS9, BCOR, BEST1, BFSP1, BFSP2, BLOC1S6, BMP4, BMP7, C2CD3, C2orf71, C5orf42, C8orf37, CA4, CABP4, CACNA1F, CACNA2D4, CC2D2A, CDH23, CDH3, CDHR1, CDON, CELSR2, CENPF, CEP104, CEP120, CEP164, CEP19, CEP250, CEP290, CEP41, CERKL, CFAP410, CHM, CHMP4B, CHRDL1, CIB2, CISD2, CLN3, CLN5, CLN6, CLN8, CLRN1, CNGA1, CNGA3, CNGB1, CNGB3, CNNM4, COL11A1, COL11A2, COL18A1, COL2A1, COL4A1, COL4A2, COL9A1, COL9A2, COL9A3, CPLANE1, CRB1, CRX, CRYAA, CRYAB, CRYBA1, CRYBA4, CRYBB1, CRYBB2, CRYBB3, CRYGB, CRYGD, CRYGS, CSPP1, CTDP1, CTSD, CTSF, CWC27, CYP1B1, CYP27A1, CYP4V2, DCDC2, DHDDS, DNAJC5, DNM1L, DTNBP1, DYNC2H1, EDNRB, EFEMP1, ELOVL4, EPHA2, ERCC1, ERCC2, ERCC5, ERCC6, ESPN, EXOC8, EXOSC2, EYA1, EYS, FALDH, FAM161A, FANCI, FBN1, FGFR1, FLVCR1, FOXC1, FOXD3, FOXE3, FOXL2, FRAS1, FREM1, FREM2, FRMD7, FSCN2, FYCO1, FZD4, GCNT2, GDF3, GDF6, GJA3, GJA8, GLA, GLI2, GLIS2, GNAT1, GNAT2, GNB3, GNPTG, GPR143, GPR179, GRIP1, GRK1, GRM6, GRN, GUCA1A, GUCA1B, GUCY2D, HADHA, HARS, HCCS, HESX1, HGSNAT, HK1, HMCN1, HMX1, HPS1, HPS3, HPS4, HPS5, HPS6, HSF4, HYLS1, IDH3B, IDS, IFT140, IFT172, IFT27, IFT43, IFT57, IFT74, IFT80, IFT81, IMPDH1, IMPG2, INPP5E, INVS, IQCB1, ITM2B, JAG1, JAM3, KARS1, KCNJ13, KCNV2, KIAA0556, KIAA0586, KIF11, KIF14, KIF3B, KIF7, KIZ, KLHL7, LAMA1, LCA5, LIM2, LMX1B, LRAT, LRIT3, LRP5, LTBP2, LYST, LZTFL1, MAF, MAK, MC1R, MERTK, MFN2, MFRP, MFSD8, MIP, MITF, MKKS, MKS1, MLPH, MTTP, MVK, MYH9, MYO5A, MYO7A, MYOC, NAA10, NBAS, NDP, NEK1, NEK8, NEUROD1, NHS, NMNAT1, NOTCH2, NPHP1, NPHP3, NPHP4, NR2E3, NR2F1, NRL, NYX, OAT, OCA2, OCRL, OFD1, OPA1, OPA3,*

		OPN1SW, OTX2, P3H2, PACS1, PANK2, PAX2, PAX3, PAX6, PCARE, PCDH15, PCYT1A, PDE6A, PDE6B, PDE6C, PDE6D, PDE6G, PDE6H, PDZD7, PEX1, PEX2, PEX6, PEX7, PGK1, PHYH, PIBF1, PIGL, PIK3R1, PISD, PITPNM3, PITX2, PITX3, PLA2G5, PLK4, PNPLA6, POC1B, POMGNT1, PORCN, PPT1, PRCD, PROKR2, PROM1, PRPF3, PRPF31, PRPF6, PRPF8, PRPH2, PRPS1, PRSS56, PSEN1, PTPN11, PXDN, PYGM, RAB18, RAB27A, RAB3GAP1, RAB3GAP2, RAX, RAX2, RB1, RBP3, RBP4, RCBTB1, RD3, RDH11, RDH12, RDH5, RGR, RGS9, RGS9BP, RHO, RIMS1, RIMS2, RLBP1, ROM1, RP1, RP1L1, RP2, RP9, RPE65, RPGR, RPGRIP1, RPGRIP1L, RS1, RTN4IP1, SAG, SALL4, SCAPER, SCLT1, SDCCAG8, SEMA3E, SEMA4A, SGSH, SHH, SIL1, SIX3, SIX6, SLC16A12, SLC24A1, SLC24A5, SLC25A46, SLC33A1, SLC41A1, SLC45A2, SLC7A14, SLC9A6, SMARCA4, SMOC1, SNAI2, SNRNP200, SNX3, SOX10, SOX2, SOX3, SPATA7, SPG7, SSBP1, STRA6, SUFU, TBX22, TCTN1, TCTN2, TCTN3, TDRD7, TEAD1, TFAP2A, TGFB2, TIMM8A, TIMP3, TLCD3B, TMEM107, TMEM114, TMEM126A, TMEM138, TMEM216, TMEM231, TMEM237, TMEM67, TOGARAM1, TOPORS, TPP1, TREX1, TRIM32, TRNT1, TRPM1, TSFM, TSPAN12, TTC21B, TTC8, TTPA, TUB, TUBGCP4, TUBGCP6, TULP1, TYR, TYRP1, UNC119, USH1C, USH1G, USH2A, VAX1, VCAN, VIM, VPS13B, VSX2, WDPCP, WDR19, WDR34, WDR35, WDR60, WFS1, WHRN, XPNPEP3, ZNF423, ZNF513
Customized aCGH[a]	aCGH	*ABCA4, ADAM9, AIPL1,* ***ALMS1****, ARL2BP, ARL6, BBS1,* ***BBS12****, BBS2, BEST1, C1QTNF5, C21ORF2, C2ORF71, C8ORF37, CA4, CACNA1F, CACNA2D4,* ***CCT2****, CDHR1,* ***CEP290****, CERKL, CFH, CLRN1,* ***CLUAP1****, CNGA1, CNGA3, CNGB1, CNGB3, CNNM4, CRB1, CRX, DHDDS, DHX38, EFEMP1, ELOVL4, EMC1, EML4, EYS, FAM161A, FSCN2, GPR125, GUCA1A, GUCA1B, GUCY2D, HGSNAT, HK1, HMCN1, IDH3B,* ***IFT140****, IFT172, IMPDH1, IMPG1, IMPG2,* ***IQCB1****,* ***KCNJ13****, KCNV2, KIAA1549, KLHL7,* ***LCA5****, LRAT, MAK, MERTK, MVK,* ***MYO7A****, NEK2, NEUROD1,* ***NMNAT1****, NR2E3, NRL, OFD1, OR2W3, PDE6A, PDE6B, PDE6C, PDE6G, PDE6H,* ***PDZD7****, PITPNM3, PLK1S1, POC1B, PRCD, PROM1, PRPF3, PRPF31, PRPF4, PRPF6, PRPF8, PRPH2, RAB28, RAX2, RBP3, RCBTB1,* ***RD3****, RDH12, RDH5, RGR, RHO, RIMS1, RLBP1, ROM1, RP1, RP1L1, RP2, RP9, RPE65, RPGR, RPGRIP1, SAG,* ***SAMD11****, SEMA4A, SF3B2, SLC7A14, SNRNP200, SPATA7, TIMP3, TOPORS, TTC8, TTLL5, TULP1, UNC119, USH2A,* ***VPS13B****,* ***WDPCP****, ZNF408, ZNF513*

aCGH: array-based comparative genomic hybridization; CES: Clinical Exome Sequencing; WES: Whole Exome Sequencing. [a] Genes in **bold** those added to the original design described in Van Cauwenberg et al. 2017 (PMID: 27608171)

Supplementary Table S3 Summary of the different *in-house* bioinformatic pipelines used for the study of Single Nucleotide Variations (SNV) and Copy Number Variations (CNV) in the WES analysis in our cohort, as well as the prioritization characteristics of each pipeline

**Bioinformatic pipeline**		**Pipeline_1**	**Pipeline_2**	**Pipeline_3**
**Aligning to GRCh37/hg19**		BWA v0.7.12-r1039	BWA v0.7.15	Novoalign software v4.02.02
**Variant calling**		GATK v3.4-46	GATK v4.0.5.1	GATK v4.1.2.0
**Variant annotation**		ANNOVAR		
**Filtering pipeline**	**Quality and coverage**	Hard filtering: my_SNP_filter: QD < 2.0, MQ < 40.0, MQRankSum < -12.5 and ReadPosRankSum < -8.0 and my_INDEL_filter: QD < 2.0 and ReadPosRankSum < -20.0<br>FILTER=PASS, DP > 10, QUAL > 100		DP > 10<br>perc_alt > 0.25<br>GQ > 30
	**Allelic frequency data**	gnomAD, NHLBI Exome Variant Server, 1000 Genomes Project, CSVS<br>MAX_AF < 0.05		ExAC, gnomAD, 1000 Genomes, NHLBI Exome Variant Server, GME Variome, ABraOM, in-house databases<br>MAX_AF < 0.01; homozygous patients in ExAC < 2
	**Predictors and evolutionary conservation of affected residue**	Implemented splicing predictors in the Alamut software<br>Predictors of the impact caused on the protein (SIFT, Polyphen2, Mutation Taster, M-CAP, CADD > 10, FATHMM, PROVEAN)<br>Gene loss of function predictors (LoFtool, ExACpLI)		Predictors of the impact caused on the protein<br>Predicted impact on messenger RNA (mRNA) splicing<br>In-house variant frequencies and splicing predictors (MaxEntScan and SpliceAI); FS < 25
	**SNPs annotation**	dbSNP		
	**HOMO_regions**	N/A		AutoMap tool (Quinodoz et al. 2021)
**CNV detection (Filters)**		CoNVaDING<br>(regionThreshold = 20, ratioCutOffLow = 0.65, ratioCutOffHigh = 1.3, zScoreCutOffLow = -3, and zScoreCutOffHigh = 3)		ExomeDepth
**Year**		2016 (vcf reannotated in 2018)	2018	2020

BWA: Burrows-Wheeler Aligner; CSVS: Collaborative Spanish Variant Server; DP: depth of coverage; FS: functional significance; GATK: Genome Analysis Toolkit; GQ: genotype quality; HOMO_regions: homozygosity regions; MAX_AF: highest allele frequency; MQ: mapping quality; N/A: non-available; perc_alt: frequency of the alternative allele; QD: quality of depth; QUAL: quality; WES: whole-exome sequencing. gnomAD/ExAC (https://gnomad.broadinstitute.org/), NHLBI Exome Variant Server (https://evs.gs.washington.edu/EVS/), 1000 Genomes Project (https://www.internationalgenome.org/), Collaborative Spanish Variant Server (https://csvs.babelomics.org/), GME Variome (https://igm.ucsd.edu/gme/index.php), and ABraOM (https://abraom.ib.usp.br/)

Supplementary Table S5 Diagnostic yield of the different molecular approaches for the study of the SRD cases, comparing the well-known syndromes (CILIOPATHY & SPECIFIC) vs the complete cohort

NGS study			Well-known syndromes diagnostic rate (characterized/total probands)	Complete cohort diagnostic rate (characterized/total probands)
**SNV (+CNV[a])**	CES	First-tier (prospective cases)	77% (10/13)	83% (15/18)
		Retrospective cases	35% (10/29)	25% (20/79)
		Total cases with CES analysis	**48% (20/42)**<br>SNV: 18; CNV: 2; SNV+CNV: 0	**36% (35/97)**<br>SNV: 32; CNV: 3; SNV+CNV: 0
	WES	447-gene subpanel analysis	50% (4/8)	30% (11/37)
		Reanalysis (including hypothesis-free approach)	50% (2/4)	19% (5/26)
		Total analysis by WES	**75% (6/8)**<br>SNV: 5; CNV: 0; SNV+CNV: 1	**43% (16/37)**<br>SNV: 15; CNV: 0 ; SNV+CNV: 1
	mtDNA sequencing		0% (0/0)	20% (1/5)
**CNV**	aCGH (60K, 400K)		0% (0/4)	13% (2/16)[b]
**TOTAL**			**% (26/42)**<br>SNV: 23; CNV: 2; SNV+CNV: 1	**52% (52/100)**<br>SNV: 45; CNV: 3; SNV+CNV: 3[b]

[a] CNV bioinformatic detection (Commercial SOPHiA DDM platform; in-house pipeline) included. [b] RP-1018 and RP-1321 (dual diagnosis) were characterized using a combination of a SNV and CNV detection techniques. aCGH: array of comparative genomic hybridization; CES: clinical exome sequencing; CNV: Copy Number Variation; mtDNA: mitochondrial DNA; NGS: Next-generation sequencing; SNV: Single Nucleotide Variation; SRD: syndromic retinal diseases; VUS: variant of uncertain significance; WES: whole-exome sequencing

Supplementary Table S6 Summary of the characterized cases through WES reanalysis and the key points that led to the diagnosis

Reanalysis	Time frame reanalysis (years)	Bioinformatic pipeline[a]	Case	Gene	Case status
New genes and associations reported in the literature since the original analysis	2	1	RP-2176	*HK1*(Okur et al. 2019)	Characterized
Hypothesis-free approach	1	2	RP-0897	*GALE*	Phenotypically partially characterized
	2	1 & 3	RP-2005	*LRRC32*	Characterized
	2	2 & 3	RP-2032	*MCOLN1*	Characterized
Compilation of new data and clinical reassessment	2	1 & 3	RP-2085	*CDH23*	Characterized

[a] Bioinformatic pipelines are described in the Supplementary Table S3. WES: Whole Exome Sequencing

Supplementary Table S9 Comparative table of published cohorts of syndromic retinal dystrophies

	**Cohort 1 (This work)**	**Cohort 2 (Jiman et al. 2020)**	**Cohort 3 (Manara et al. 2019)**	**Cohort 4 (Shaheen et al. 2016)**
**Clinical composition**	Miscellanea of SRD	Miscellanea of SRD	BBS	Syndromic ciliopathy spectrum
**Consanguinity**	+/-	N/A	-	+
**Study approach**	Singleton	Singleton	Singleton	Singleton and familiar
**NGS technique**	Various	Customized panel	Customized panel	Various
**Total or partially phenotypically characterized families (n)**	52	58	12	225
**Uncharacterized families (n)**	48	48	8	40
**Characterization rate**	52%	55%	60%	85%

Chi-square test of independence with a significance level of 0.05:

- <u>Cohort 1-4</u>. X^2 (3, N= 491) = 56.8418, p <0.0001. There is a significant difference between groups 1-4, so the proportion of characterized patients differ by studies.
- <u>Cohort 1-3</u>. X^2 (2, N= 226) = 0.4728, p = 0.789478. The statistical test showed that there is no significant difference between characterization rates in the studies 1-3.

Statistical analysis using chi-square test with a significance level of p < 0.05. BBS: Bardet-Biedl syndrome; N/A: non-available; SRD: syndromic retinal diseases; +: yes; -: no; +/-: in some cases

Supplementary Table S10 Comparative table with different reanalysis studies

Study	Year	Number of included cases	Diseases	Time frame since 1st analysis	Diagnostic yield of the reanalysis	Technique	Reanalysis method
(Wenger et al. 2017)	2017	40 individuals	Miscellanea	20 months	10% (4/40)	Clinical exome	Bioinformatic and literature
(Ewans et al. 2018)	2018	54 patients (of 37 families)	Miscellanea	12 months	15% (4/26)	WES (singleton and trios)	Clinical revaluation, bioinformatic and literature
(Wright et al. 2018)	2018	1133 families	Developmental disorders	3 years	Increased by 13%	WES (trios)	Bioinformatic and literature
(Jalkh et al. 2019)	2019	200 patients	Miscellanea	2 years	6.5% (13/101)	WES (singleton)	Bioinformatic and literature
(Li et al. 2019)	2019	76 families	Epilepsy and ID/MR	6 to >12 months	10.5% (8/76)	WES (trios)	Clinical revaluation, bioinformatic and literature
(Liu et al. 2019)	2019	250 patients	Miscellanea	5 years	Increased from 24.8% to 46.8%	WES (singleton)	Bioinformatic and literature ("manual")
(Liu et al. 2019)	2019	2000 cases	Miscellanea	4 years	Increased from 25.2% to 36.7%	WES (singleton)	Bioinformatic and literature (semi-automated)
**This work**	2021	100 cases	SRD	1-3 years	19% (5/26)	Customized panel, clinical exome, WES (singleton)	Clinical revaluation, bioinformatic and literature

ID: intellectual disability; MR: mental retardation; SRD: syndromic retinal diseases; WES: Whole-Exome Sequencing

Supplementary Fig. S1 Working flowchart of the molecular study of the SRD cases of our cohort.

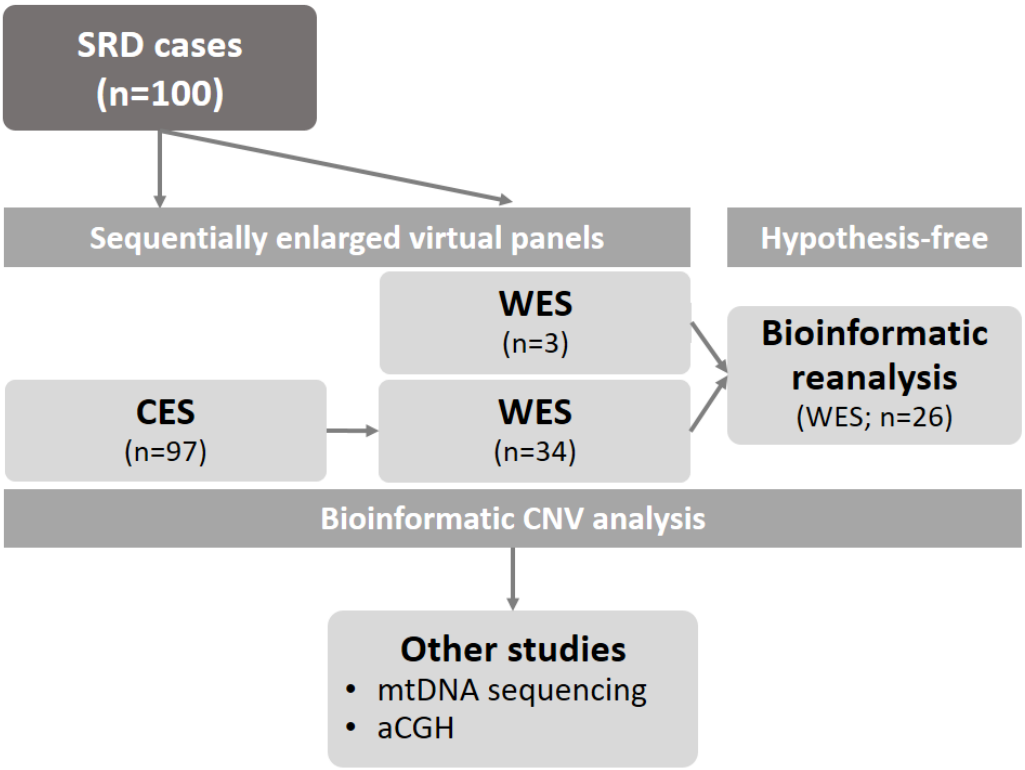

Cases have been studied by CES and/or WES, using sequential increased sizes of gene subpanels: 1) 136-gene subpanel (minimal virtual panel); 2) enlarged virtual panels of 241 and 447 genes; and 3) hypothesis-free approach. Each step of the flowchart included the bioinformatic CNV analysis of the data. In cases with suspected mtDNA disorder, mtDNA sequencing was performed. aCGH: array of comparative genomic hybridization; CES: clinical exome sequencing; CNV: Copy Number Variation; mtDNA: mitochondrial DNA; SNV: Single Nucleotide Variation; SRD: syndromic retinal diseases; WES: whole-exome sequencing

Supplementary Fig. S2 Distribution of cases after the molecular studies.

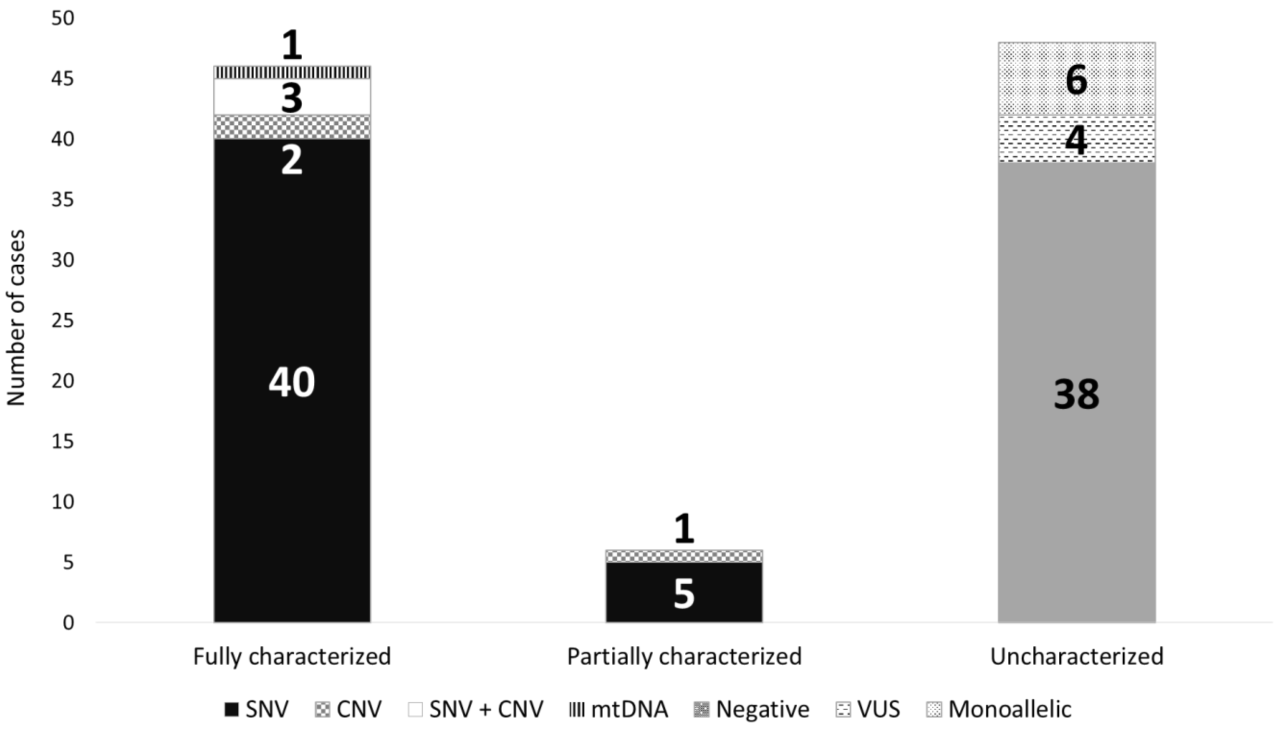

Cases were classified according to the genetic results and their type (SNV, CNV, SNV+CNV and mitochondrial DNA. CNV: Copy Number Variation; mtDNA: mitochondrial DNA; SNV: Single Nucleotide Variation

Supplementary Fig. S3 Variant analysis of the characterized and likely pathogenic/VUS monoallelic cases.

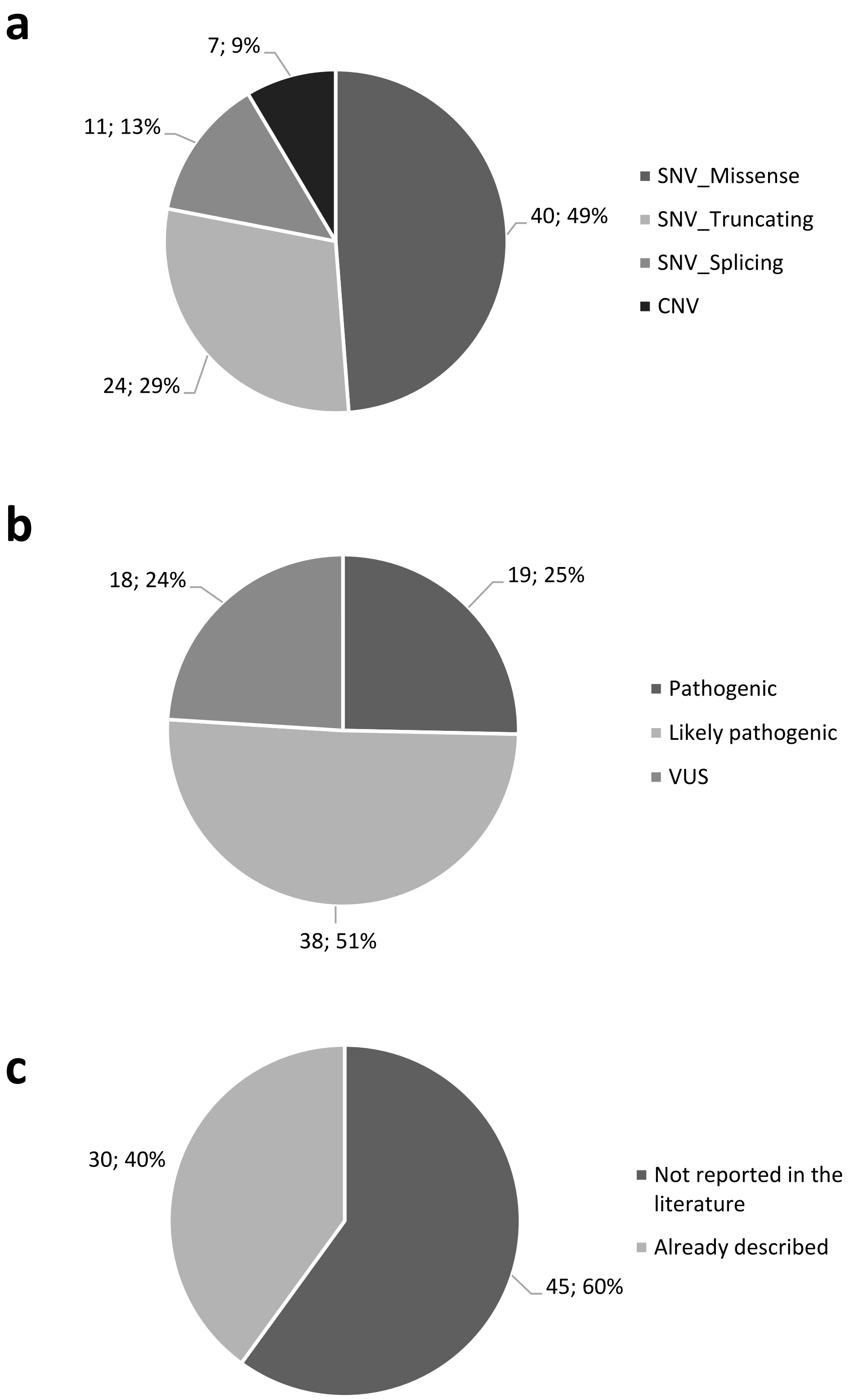

(a) Distribution of the variants. Variants could be Single Nucleotide Variations (SNVs) (missense, truncating or splicing) or Copy Number Variations (CNVs). **(b)** SNV pathogenicity distribution. Applying the guidelines of the American College of Medical Genetics and Genomics (ACMG) and the European Society of Human Genetics (ESHG), variants could be classified regarding their pathogenicity into pathogenic, likely pathogenic or variant of uncertain significance (VUS). **(c)** Distribution of new and already reported SNV variants

Supplementary Fig. S4 Dual diagnosis in RP-1018 by targeted NGS and array of comparative genomic hybridization (aCGH).

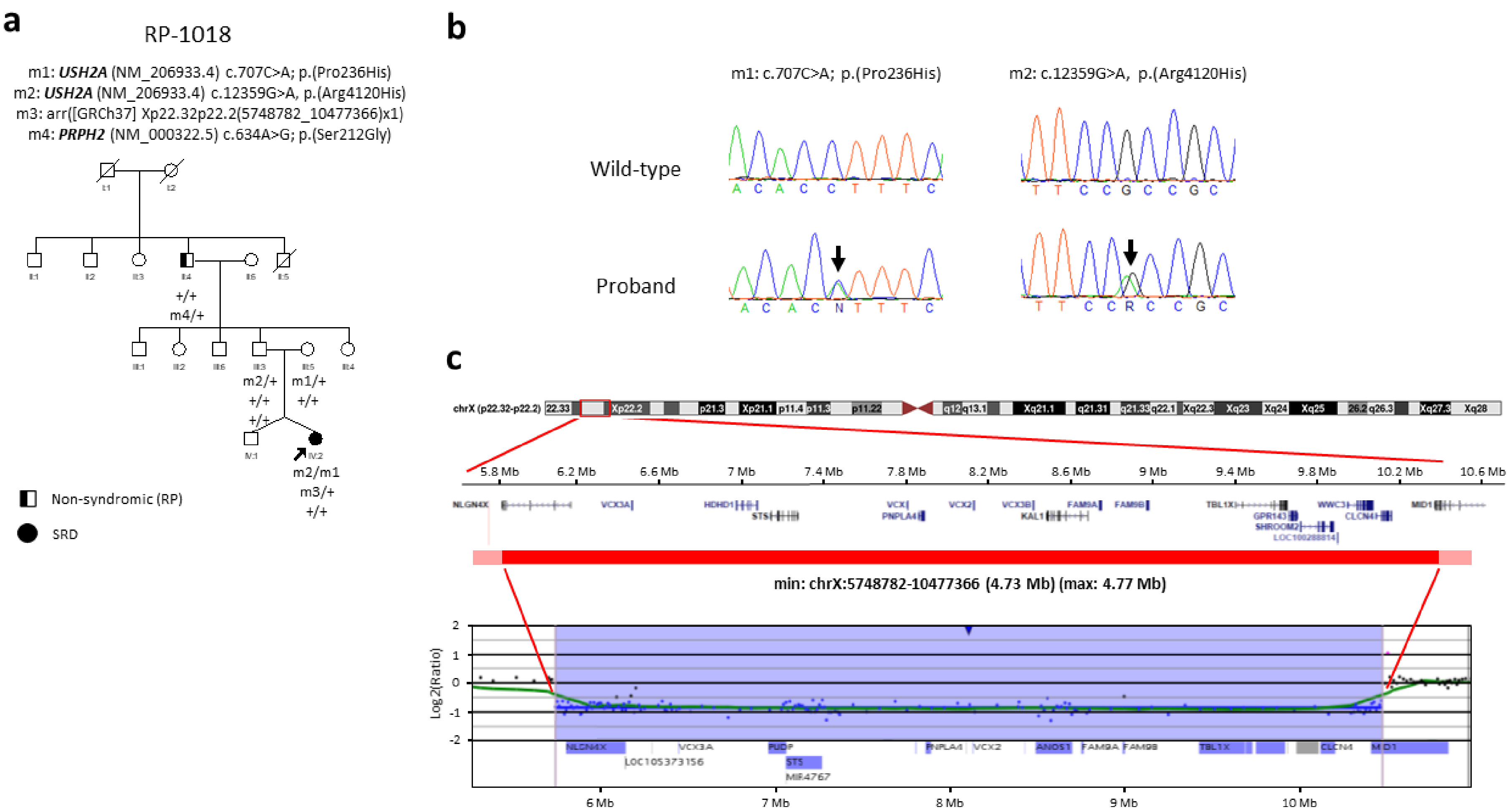

(a) Pedigree and segregation analysis within the family. Genetic findings were segregated in all the available family members and indicated with "+" for wild-type alleles and "m1, m2, m3 and m4" for each mutated allele. RP: retinitis pigmentosa; SRD: syndromic retinal disease. **(b)** Electropherograms of the two heterozygous variants in *USH2A* for wild-type and mutated allele. **(c)** Genomic rearrangement on Xq22.32-22.2 region. The presence of a 4.73 Mb de novo deletion in chromosome X was observed within Xq22.32-22.2 (minimum genomic coordinates: chrX:5748782-10477366) (dark red), involving 16 genes (*NLGN4X, VCX3A, PUDP, STS, VCX, PNPLA4, VCX2, VCX3B, ANOS1, FAM9A, FAM9B, TBL1X, GPR143, SHROOM2, CLCN4* and *MID1*), of which six were in OMIM, standing out *NLGN4* and *MID1*, associated to Mental retardation, X-linked (MIM #300495) and Opitz GBBB syndrome, type I (MIM #300000), respectively. The horizontal axis shows the genomic position along the genome (GRCh37 – hg19) and the vertical axis the log2 ratio values (-2/-1: deletion; 0: normal pattern; 1/2: duplication)

Supplementary Fig. S5 Dual diagnosis in RP-1321 by targeted NGS and array of comparative genomic hybridization (aCGH).

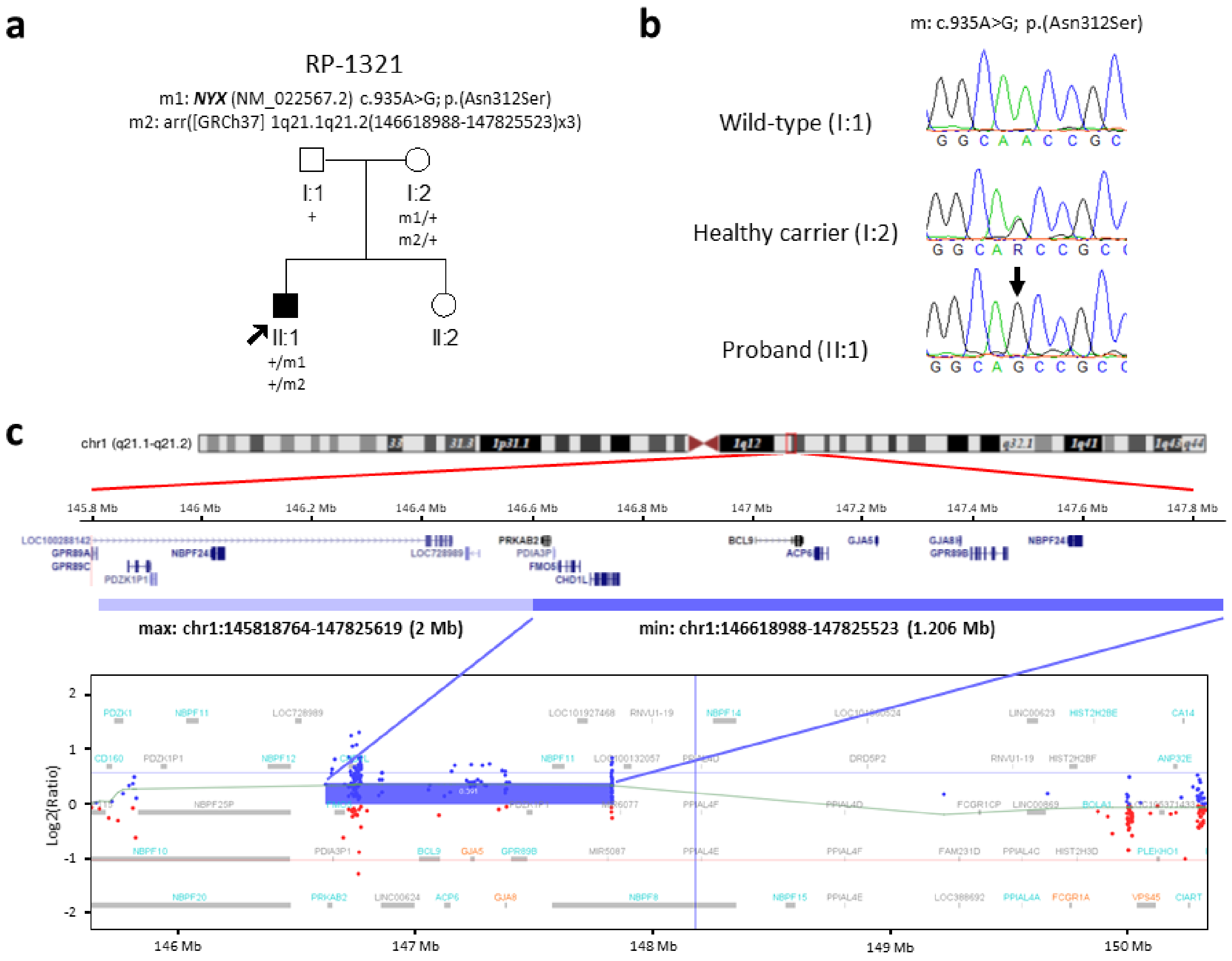

(a) Pedigree and segregation analysis within the family. Genetic findings were segregated in all the available family members and indicated with "+" for wild-type alleles and "m1 and m2" for each mutated allele. **(b)** Electropherograms of the *NYX* variant for wild-type, heterozygous carrier and hemizygous. **(c)** Genomic rearrangement on 1q21.1q21.2 region. A minimum 1.206 Mb duplication (genomic coordinates: chr1:146618988-147825523) (dark blue) and maximum of 2 Mb (genomic coordinates: chr1:145818764-147825619) (light blue) was observed using aCGH. This duplication comprised 13 genes (*FMO5*, *CHD1L*, *BCL9*, *GJA5*, *GJA8*, *PRKAB2*, *PDIA3P*, *ACP6*, *GPR98B*, *GPR98C*, *PDZK1P1*, *NBPF11* and *NBPF24*), of which 7 are included in OMIM. The horizontal axis shows the genomic position along the genome (GRCh37 – hg19) and the vertical axis the log2 ratio values (-2/-1: deletion; 0: normal pattern; 1/2: duplication

References

den Boer MEJ, Wanders RJA, Morris AAM, et al (2002) Long-chain 3-hydroxyacyl-CoA dehydrogenase deficiency: clinical presentation and follow-up of 50 patients. Pediatrics 109:99–104. https://doi.org/10.1542/peds.109.1.99

Ewans LJ, Schofield D, Shrestha R, et al (2018) Whole-exome sequencing reanalysis at 12 months boosts diagnosis and is cost-effective when applied early in Mendelian disorders. Genet Med 20:1564–1574. https://doi.org/10.1038/gim.2018.39

Forsythe E, Beales PL (2013) Bardet-Biedl syndrome. Eur J Hum Genet 21:8–13. https://doi.org/10.1038/ejhg.2012.115

Jalkh N, Corbani S, Haidar Z, et al (2019) The added value of WES reanalysis in the field of genetic diagnosis: lessons learned from 200 exomes in the Lebanese population. BMC Med Genomics 12:11. https://doi.org/10.1186/s12920-019-0474-y

Jiman OA, Taylor RL, Lenassi E, et al (2020) Diagnostic yield of panel-based genetic testing in syndromic inherited retinal disease. Eur J Hum Genet 28:576–586. https://doi.org/10.1038/s41431-019-0548-5

Karlberg N, Jalanko H, Perheentupa J, Lipsanen-Nyman M (2004) Mulibrey nanism: clinical features and diagnostic criteria. J Med Genet 41:92–98. https://doi.org/10.1136/jmg.2003.014118

Kashtan CE (1993) Alport Syndrome. In: Adam MP, Ardinger HH, Pagon RA, et al. (eds) GeneReviews®. University of Washington, Seattle, Seattle (WA)

Kohlschütter A, Schulz A, Bartsch U, Storch S (2019) Current and Emerging Treatment Strategies for Neuronal Ceroid Lipofuscinoses. CNS Drugs 33:315–325. https://doi.org/10.1007/s40263-019-00620-8

Li J, Gao K, Yan H, et al (2019) Reanalysis of whole exome sequencing data in patients with epilepsy and intellectual disability/mental retardation. Gene 700:168–175. https://doi.org/10.1016/j.gene.2019.03.037

Liu P, Meng L, Normand EA, et al (2019) Reanalysis of Clinical Exome Sequencing Data. N Engl J Med 380:2478–2480. https://doi.org/10.1056/NEJMc1812033

Manara E, Paolacci S, D'Esposito F, et al (2019) Mutation profile of BBS genes in patients with Bardet-Biedl syndrome: an Italian study. Ital J Pediatr 45:72. https://doi.org/10.1186/s13052-019-0659-1

Marshall JD, Beck S, Maffei P, Naggert JK (2007) Alström syndrome. Eur J Hum Genet 15:1193–1202. https://doi.org/10.1038/sj.ejhg.5201933

Nozu K, Nakanishi K, Abe Y, et al (2019) A review of clinical characteristics and genetic backgrounds in Alport syndrome. Clin Exp Nephrol 23:158–168. https://doi.org/10.1007/s10157-018-1629-4

Okur V, Cho MT, van Wijk R, et al (2019) De novo variants in HK1 associated with neurodevelopmental abnormalities and visual impairment. Eur J Hum Genet 27:1081–1089. https://doi.org/10.1038/s41431-019-0366-9

Parisi MA, Doherty D, Chance PF, Glass IA (2007) Joubert syndrome (and related disorders) (OMIM 213300). Eur J Hum Genet 15:511–521. https://doi.org/10.1038/sj.ejhg.5201648

Quinodoz M, Peter VG, Bedoni N, et al (2021) AutoMap is a high performance homozygosity mapping tool using next-generation sequencing data. Nat Commun 12:518. https://doi.org/10.1038/s41467-020-20584-4

Shaheen R, Szymanska K, Basu B, et al (2016) Characterizing the morbid genome of ciliopathies. Genome Biol 17:242. https://doi.org/10.1186/s13059-016-1099-5

Tranebjærg L, Barrett T, Rendtorff ND (1993) WFS1 Wolfram Syndrome Spectrum Disorder. In: Adam MP, Ardinger HH, Pagon RA, et al. (eds) GeneReviews®. University of Washington, Seattle, Seattle (WA)

Wang H, Falk MJ, Wensel C, Traboulsi EI (1993) Cohen Syndrome. In: Adam MP, Ardinger HH, Pagon RA, et al. (eds) GeneReviews®. University of Washington, Seattle, Seattle (WA)

Wenger AM, Guturu H, Bernstein JA, Bejerano G (2017) Systematic reanalysis of clinical exome data yields additional diagnoses: implications for providers. Genet Med 19:209–214. https://doi.org/10.1038/gim.2016.88

Wright CF, McRae JF, Clayton S, et al (2018) Making new genetic diagnoses with old data: iterative reanalysis and reporting from genome-wide data in 1,133 families with developmental disorders. Genet Med 20:1216–1223. https://doi.org/10.1038/gim.2017.246
